# A Review of Relative Pollen Productivity Estimates From Temperate China for Pollen-Based Quantitative Reconstruction of Past Plant Cover

**DOI:** 10.3389/fpls.2018.01214

**Published:** 2018-09-05

**Authors:** Furong Li, Marie-José Gaillard, Qinghai Xu, Mairi J. Bunting, Yuecong Li, Jie Li, Huishuang Mu, Jingyao Lu, Panpan Zhang, Shengrui Zhang, Qiaoyu Cui, Yahong Zhang, Wei Shen

**Affiliations:** ^1^Department of Biology and Environmental Science, Linnaeus University, Kalmar, Sweden; ^2^College of Resources and Environment Science, Hebei Normal University, Shijiazhuang, China; ^3^School of Environmental Sciences, University of Hull, Hull, United Kingdom; ^4^Institute of Geographic Sciences and Natural Resources Research, Chinese Academy of Sciences, Beijing, China; ^5^Institute of Nihewan Archaeology, Hebei Normal University, Shijiazhuang, China

**Keywords:** Extended R-Value (ERV) model, relevant source area of pollen (RSAP), fall speed of pollen (FSP), vegetation-data collection, modern pollen sampling

## Abstract

Model-based quantitative reconstruction of past plant cover in Europe has shown great potential for: (i) testing hypotheses related to Holocene vegetation dynamics, biodiversity, and their relationships with climate and land use; (ii) studying long term interactions between climate and land use. Similar model-based quantitative reconstruction of plant cover in China has been restricted due to the lack of standardized datasets of existing estimates of relative pollen productivity (RPP). This study presents the first synthesis of all RPP values available to date for 39 major plant taxa from temperate China and proposes standardized RPP datasets that can be used for model-based quantitative reconstructions of past plant cover using fossil pollen records for the region. We review 11 RPP studies in temperate China based on modern pollen and related vegetation data around the pollen samples. The study areas include meadow, steppe and desert vegetation, various woodland types, and cultural landscapes. We evaluate the strategies of each study in terms of selection of study areas and distribution of study sites; pollen- and vegetation-data collection in field; vegetation-data collection from satellite images and vegetation maps; and data analysis. We compare all available RPP estimates, select values based on precise rules and calculate mean RPP estimates. We propose two standardized RPP datasets for 31 (Alt1) and 29 (Alt2) plant taxa. The ranking of mean RPPs (Alt-2) relative to Poaceae (= 1) for eight major taxa is: *Artemisia* (21) > *Pinus* (18.4) > *Betula* (12.5) > *Castanea* (11.5) > Elaeagnaceae (8.8) > *Juglans* (7.5) > Compositae (4.5) > Amaranthaceae/Chenopodiaceae (4). We conclude that although RPPs are comparable between Europe and China for some genera and families, they can differ very significantly, e.g., *Artemisia*, Compositae, and Amaranthaceae/Chenopodiaceae. For some taxa, we present the first RPP estimates e.g. *Castanea*, Elaeagnaceae, and *Juglans*. The proposed standardized RPP datasets are essential for model-based reconstructions of past plant cover using fossil pollen records from temperate China.

## Introduction

Pollen-based quantitative reconstructions of past plant cover may be required to answer specific questions related to past vegetation changes and/or the interactions between plant cover, humans, and climate. Although pollen percentages and pollen accumulation rates (PARs) have been widely used to estimate past vegetation cover at the local to global scales using various approaches such as multi-proxy studies (e.g., Berglund, 1991) and biomization (e.g., Prentice and Jolly, 2000; Williams et al., 2008; Ni et al., 2010, 2014), these methods do not allow reconstructions of plant abundance in absolute values (in % cover of the total land for a specified area or m^2^/m^2^) for individual plant taxa. Although the cover of tree versus herb vegetation at a continental to global scale can roughly be estimated using biomization (e.g., Davis et al., 2015) and pseudo-biomization (e.g., Fyfe et al., 2015), it has been shown by comparison of satellite-derived with pollen-based estimates of tree cover in e.g., northern Asia that pollen-inferred tree cover is often too high for most tree categories, largely due to long-distance transport of pollen (Tarasov et al., 2007). In addition, these methods cannot provide details on the respective proportions of plant taxa, plant groups such as conifers, broad-leaved trees, and herbs, or land units such as forest, grassland, and cultivated land. So far, the soundest approaches for obtaining estimates of plant cover at the taxon level are the Landscape Reconstruction Algorithm (LRA) of Sugita (2007a,b) and the Multiple Scenario Approach (MSA) of Bunting and Middleton (2009). In order to use the LRA or the MSA, a number of parameters need to be known, including the pollen productivity of the taxa of interest. The most common method presently used for estimating relative pollen productivity (RPP) is the application of the Extended R-Value (ERV) model (Parsons and Prentice, 1981; Prentice and Parsons, 1983) on modern data sets of pollen assemblages and related vegetation data. A relatively large number of RPP estimates have been produced in Europe using this method (see reviews by Broström et al., 2008; Mazier et al., 2012; Bunting et al., 2013) and northern America (e.g., Calcote, 1995; Commerford et al., 2013).

The LRA corrects biases in the pollen representation of vegetation due to inter-taxonomic differences in pollen productivity and dispersal ability and to variations in the size and type of the basin where the record accumulates (lake, bog). It is a two-step process, beginning with the REVEALS model (Sugita, 2007a) which estimates regional vegetation composition using pollen records from large lakes. Then the LOVE model (Sugita, 2007b) can be used to reconstruct local plant abundance using pollen records from small sites. REVEALS and LOVE have been tested and validated for modern and historical time landscapes in many parts of Europe and northern America (e.g., Hellman et al., 2008a,b; Sugita et al., 2010; Cui et al., 2014; Hjelle et al., 2015; Trondman et al., 2016). In Europe, REVEALS-based maps (Pirzamanbein et al., 2014; Trondman et al., 2015) or time series (e.g., Nielsen et al., 2012; Fyfe et al., 2013; Marquer et al., 2014) of past land cover and landscape openness were shown to be appropriate for climate modeling (Strandberg et al., 2014) and analyses of past changes in vegetation composition related to human impact and climate (Marquer et al., 2017). Similarly, the LOVE model was shown to perform well in estimating plant cover at the local spatial scale, providing insights that neither pollen percentages nor PARs could offer (e.g., Fredh et al., 2012; Cui et al., 2014). Nonetheless, the application of REVEALS and LOVE is still very limited compared to narrative interpretation of pollen records that remains dominant in the literature. This is largely due to the lack of values of pollen productivity in many regions of the world.

**Figure 1:**
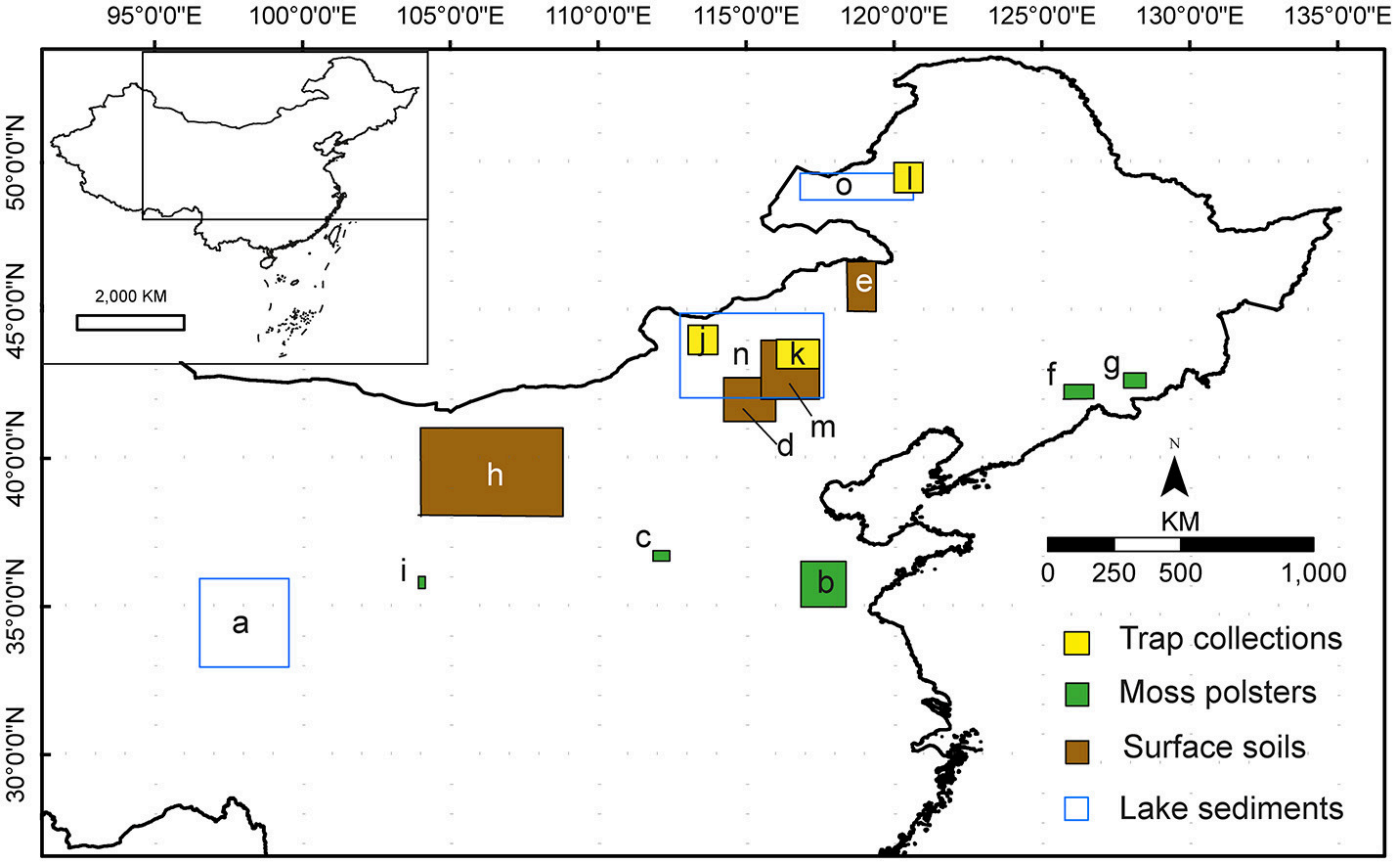
Location of the areas selected for the 11 studies on relative pollen productivities (RPP) reviewed in this paper. a. (Wang and Herzschuh, 2011), Tibetan Plateau. b. (Li et al., 2017), Shandong. c. (Zhang et al., 2017), Taiyue Mountains. d. (Ge et al., 2015), central Inner Mongolia. e. Li et al., manuscript in preparation, northeastern Inner Mongolia. f. (Li et al., 2015), Changbai Mountains. g. (Zhang et al., 2017), Changbai Mountains. h. (Li et al., 2011), Alashan Plateau, western Inner Mongolia. i. (Wu et al., 2013), Xinglong Mountains. J. (He et al., 2016). Hulunbeier, northeastern Inner Mongolia. k. (He et al., 2016) Xilinhaote, central Inner Mongolia; l. (He et al., 2016). Sunitezuoqi, central Inner Mongolia. m. (Xu et al., 2014), central Inner Mongolia. n. (Han et al., 2017) Xilinguole, central Inner Mongolia. o. (Han et al., 2017). Hulunbeier, northeastern Inner Mongolia.

Many research questions of interest to palaeoecologists require quantified plant cover values (e.g., Gaillard et al., 2010; Fredh et al., 2012; Cui et al., 2014; Marquer et al., 2014, 2017). For instance, quantitative reconstructions of land cover world-wide are required to test scenarios of past anthropogenic land-cover change [ALCC; e.g., KK, Kaplan et al. (2009) and HYDE, Klein Goldewijk et al. (2011)] and provide reliable, global land-cover descriptions for climate modelers (Gaillard et al., 2010). A collective effort to provide a first global reconstruction is taking place through the PAGES LandCover6k initiative (http://pastglobalchanges.org/ini/wg/landcover6k/intro; Gaillard et al., 2015). China is one of the regions of the world (with northern America, Latin America, large parts of Africa, Europe, and India) where human population growth has been particularly significant over the Holocene and had a strong impact on past land use and land cover. Pollen-based reconstruction using the REVEALS model is an important component of the PAGES LandCover6k initiative in China. In recent years, a number of RPP measurement studies have been initiated in the temperate zones of China (Li et al., 2011, 2015, 2017; Wang and Herzschuh, 2011; Wu et al., 2013; Xu et al., 2014; Ge et al., 2015; He et al., 2016; Han et al., 2017; Zhang et al., 2017; Li et al., in preparation) (Figure 1), and have supported limited application of REVEALS to reconstruct regional land cover (Wang and Herzschuh, 2011; Xu et al., 2014, 2016). In order to reconstruct land cover for the whole of temperate China, the first step is to review and evaluate the RPP estimates obtained so far, and define RPP datasets that can be used across the region following the approach of Mazier et al. (2012) in Europe. This paper presents that review, describes two alternative synthesized RPP datasets suitable for application in reconstruction, and makes recommendations on methods and target plant species for future RPP studies.

## Study areas

The studies reviewed in this paper are located in northern China (Figure 1). Descriptions of the study areas in terms of vegetation types and flora are found in the Supplementary Material. Other metadata for the study areas are presented in Tables S1, S2.

**Figure 2:**
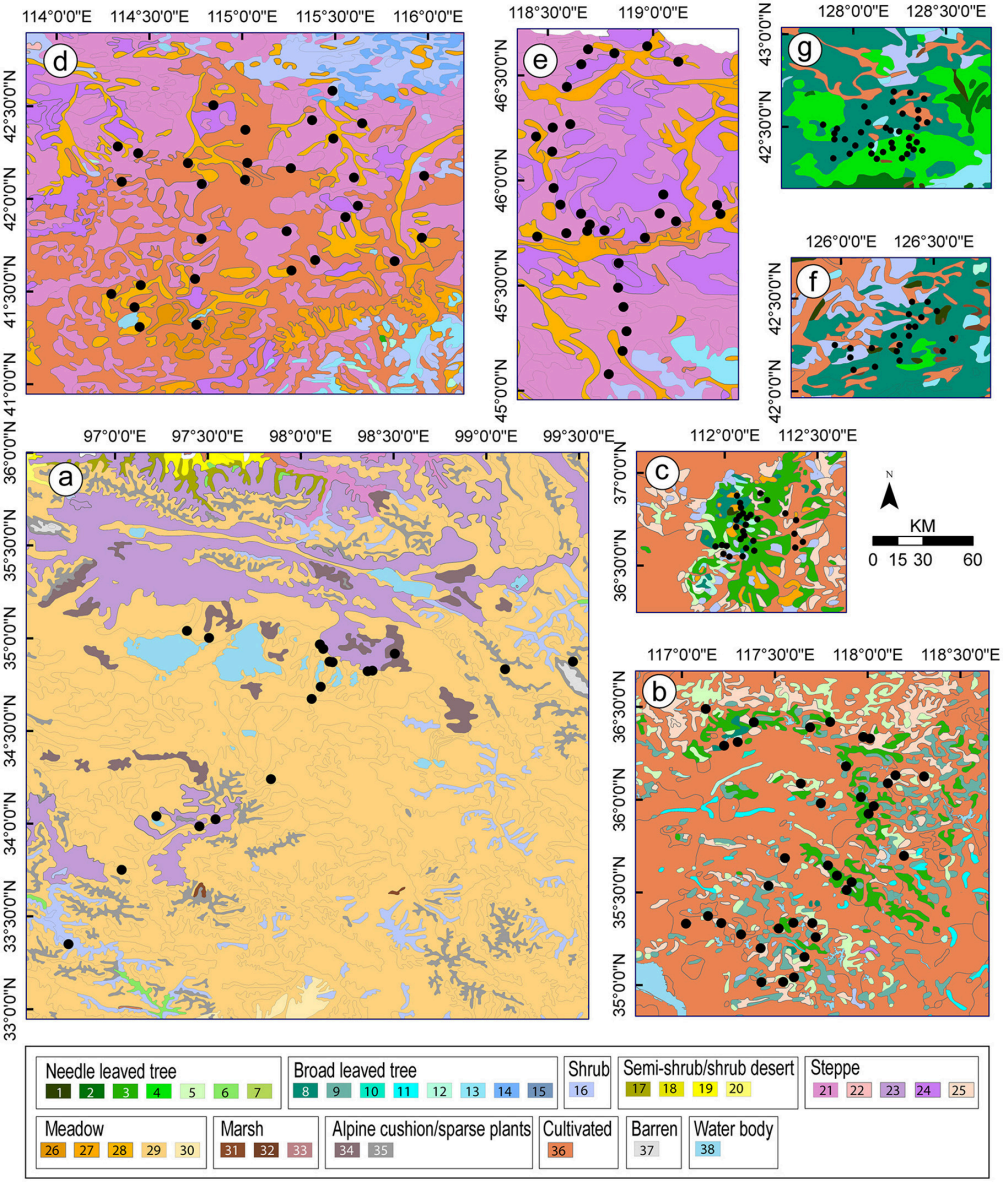
Vegetation maps for the studies (**a-g**, see Figure 1 for names of the study areas) from which values of relative pollen productivity (RPP) were selected for the synthesized RPP datasets presented in this paper (Table 3; Figure 4). Community types: 1. *Larix* spp. forest; 2. *Picea* spp. forest; 3*. Pinus* spp. forest; 4. *Pinus* spp., *Ulmus* spp. and *Fraxinus* spp. mixed forest; 5. *Platycladus orientalis* forest; 6. *Sabina* spp. forest; 7. *Larix* spp. and *Quercus* spp. *mixed* forest; 8. *Quercus* spp. forest; 9. *Robinia pseudoacacia* forest; 10. *Malus sieversii* forest; 11. *Salix* spp. forest; 12. Pure *Populus* spp. or *Populus* spp.- dominated mixed forest; 13*. Betula* spp. forest; 14. *Ulmus* spp. sparse forest; 15. *Castanopsis orthacantha* forest; 16. Temperate deciduous shrub; 17. Shrub desert; 18. Steppe desert; 19.Semi-shrub or low shrub desert; 20. *Haloxylon ammodendron* desert; 21. Temperate Poaceae-dominated mixed steppe; 22.Temperate Poaceae-dominated steppe; 23.Temperate desert steppe; 24. Poaceae-and Cyperaceae-dominated alpine steppe; 25. Poaceae-dominated shrub steppe; 26. Poaceae, *Carex* and other herbs marsh meadow; 27. Mixed halophytes meadow; 28. Cyperaceae-dominated meadow; 29. Poaceae-dominated meadow; 30. P*olygonum-*dominated meadow; 31. *Carex* marsh; 32. *Phragmites communis* marsh; 33. Alpine cushion plants; 34. Alpine sparse shrubs; 35. *Sphagnum* marsh; 36. Cultivated vegetation; 37. Barren; 38. Water body.

**Figure 3:**
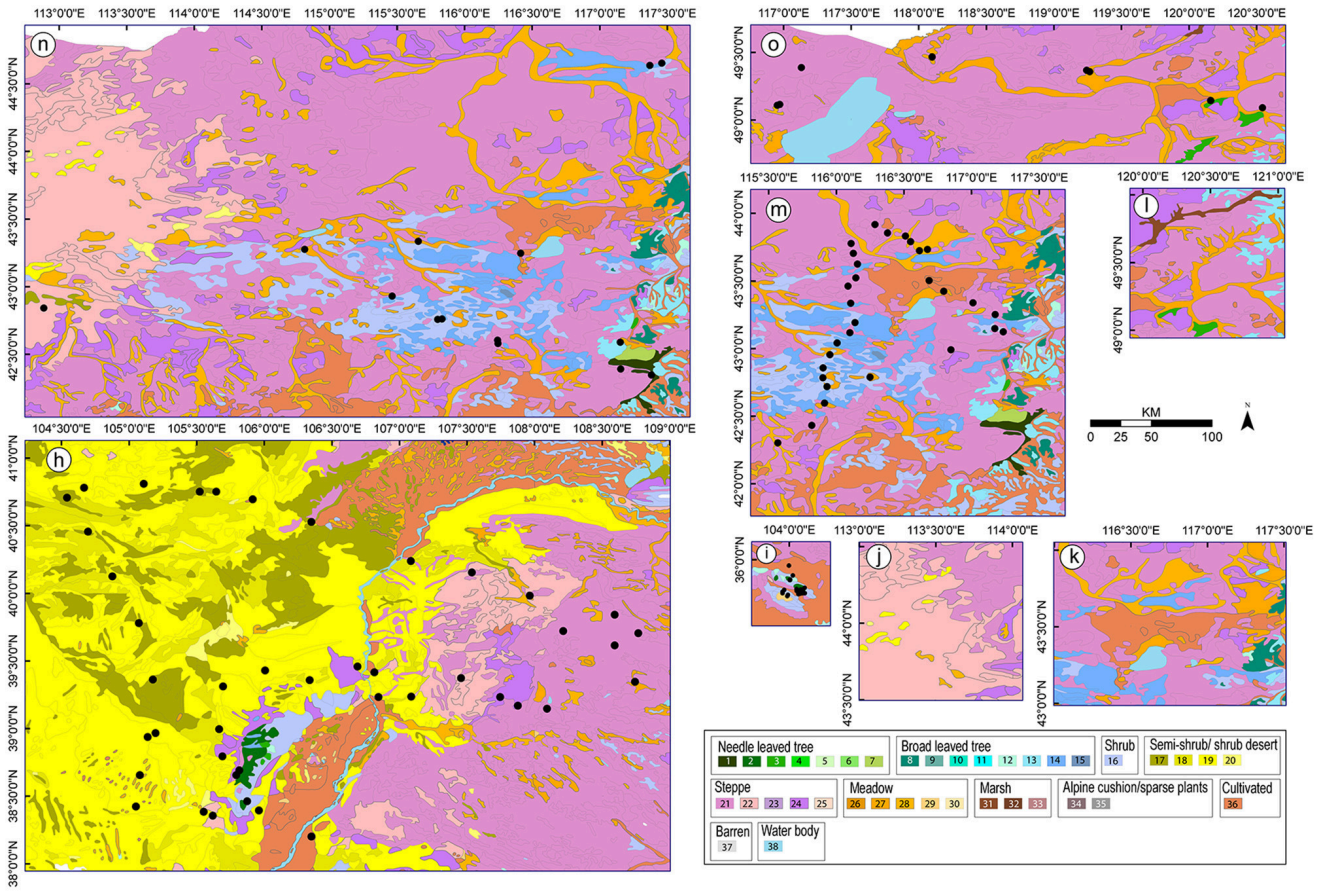
Vegetation maps for the studies (**h-o**, see Figure 1 for names of the study areas) from which values of relative pollen productivity (RPP) were not selected for the synthesized RPP datasets presented in this paper. Community types: see caption of Figure 2.

The choice of the study areas by the authors of the reviewed studies was motivated by their understanding of the environments represented in both existing palaeocological records and those to be collected in future studies. All the studies form part of larger investigations aiming to reconstruct past vegetation cover in quantitative terms (actual cover of major taxa) in order to answer questions about long-term vegetation dynamics and past climate change. This focused efforts on obtaining values of relative pollen productivity (RPP) for the major pollen taxa characteristic of forest, meadow, steppe, semi-desert, and desert communities and for key taxa indicative of human disturbance in the form of traditional cultivation (Figures 1–3; Tables S1, S2).

## Methods

### The ERV model's constraints on pollen-vegetation data for calculation of relative pollen productivity

All studies reviewed present Relative Pollen Productivity (RPP) values estimated from modern pollen and related vegetation data using the Extended R-Value (ERV) model. A full account of the ERV model and its developments is found in e.g., Bunting et al. (2013). Details of the methodological choices made for each of the 11 RPP studies reviewed in this paper are provided in the Supplementary Material. Methods varied between studies particularly in terms of site selection strategy (degree of randomness), vegetation survey methods for vegetation close to sampling points, and extraction of vegetation data for the wider area from vegetation maps or satellite pictures. Below we describe the major requirements that the modern pollen and related vegetation data should meet given the theoretical framework and assumptions of the ERV model:

- The study region should be characterized by homogenous regional vegetation, i.e., size and distribution of patches of vegetation types/land-use units should be as consistent as possible throughout the study region, and regions including major vegetation-zone boundaries and ecotones should be avoided (see also discussion). Homogeneous vegetation at the regional spatial scale ensures that the “background pollen” is consistent between all pollen sampling sites within the region, which is an assumption of the ERV model. The background pollen is defined as the pollen coming from beyond the relevant source area of pollen (RSAP) of the site (moss polster, lake, bog, etc.; Sugita, 1994). The RSAP *sensu* Sugita (1994) is defined as the distance from the pollen site beyond which the relationship between pollen and vegetation as expressed by the ERV model does not improve.
- Sites for collection of pollen and vegetation data should be of “sufficient” number and randomly distributed to meet the requirements of the ERV model and achieve a reliable estimate of the RSAP. Broström et al. (2005) have shown how important random site distribution is for the maximum likelihood method (see methods in the Supplementary Material). Non-randomly selected sites often lead to unexpected behavior of the log-likelihood curve and uncertain identification of the RSAP distance (see requirement iii). A “sufficient” number of sites is defined as, at a minimum, double the number of plant taxa for which RPP will be estimated (Sugita, personal communication; e.g., 30 sites for 15 taxa). However, the exact number of taxa for which datasets suitable for calculation of RPP will be generated is not known in advance of data collection. In most studies performed in Europe and elsewhere, the strategy has been to assume that a maximum of 15 to 20 taxa will be appropriate for ERV-model analysis and, therefore, 30 to 40 sites were used for collection of pollen and vegetation data in field (e.g., Broström et al., 2008; Mazier et al., 2012).
- Theoretically, the most reliable estimate of RPP using the ERV model should correspond to the RPP value obtained for the distance from the pollen sample corresponding to the radius of the RSAP. Therefore, one has to assume the RSAP radius and collect vegetation data over a distance larger than the assumed RSAP radius to ensure that the pollen-vegetation data will be adequate for the ERV-model calculation of the RSAP radius (Bunting et al., 2013). The assumed RSAP can be inferred from comparison with calculated RSAPs in other studies performed in comparable landscapes, or can be derived from simulation using hypothetical landscapes or existing vegetation maps (e.g., Mazier et al., 2008; Hellman et al., 2009a,b).

### Comparison and evaluation of RPP values: developing a synthesized RPP dataset for model applications

In order to compare the RPP values between studies, it is necessary that the reference taxon is the same in all studies. Therefore, a common reference taxon had to be chosen. In theory, any taxon can serve as the reference taxon. For good results, however, the reference taxon needs to be present in both pollen and vegetation data from as many sites as possible, have a wide range of values of both pollen and vegetation, and have intermediate pollen production (Bunting et al., 2013). Poaceae has often been used as the reference taxon in semi open and open landscapes, because it is the only taxon that is always present and generally has a good gradient of pollen/vegetation relationship. In forested landscapes, it has been more common to use *Quercus* or *Pinus* as reference taxa. We acknowledge that Poaceae is not an ideal reference taxon since it includes a large number of species, and the species may differ between study areas. This may result in between-site differences in RPPs for the studied taxa due to differences in Poaceae pollen production resulting from differences between the Poaceae species mixtures even though all other taxa retain the same pollen productivity. Most taxa for which RPP are calculated, *Pinus* and *Quercus* included, do include different species depending on the vegetation type and geographical location. Therefore, whatever taxon is used as reference, there will always be possible differences in RPP between studies due to different mix of species involved in each taxon. Poaceae has the advantage of being one of the few taxa that is usually common in most vegetation types of the world and represented in both vegetation and pollen data in most samples. There is so far no alternative taxon that can be used for comparison of RPPs between studies performed in different regions and continents, and in both open/semi-open and forested landscapes.

Poaceae was originally selected as the reference taxon in six of the 11 reviewed studies, while *Artemisia*, Chenopodiaceae or *Quercus* was selected for the other five studies. In order to compare the RPP values between studies, the RPPs relative to Chenopodiaceae (RPP_Chenopodiaceae_; Li et al., 2011), *Quercus* (RPP_*Quercus*_; Wu et al., 2013; Li et al., 2015; Zhang et al., 2017) and *Artemisia* (RPP_*Artemisia*_; He et al., 2016) were converted to values relative to Poaceae (RPP_Poaceae_). In the case of the study by Li et al. (2015), Poaceae was not included in the taxa selected for calculation of the RPP estimates. Therefore, we applied the method proposed by Mazier et al. (2012) to convert RPP_*Quercus*_ to RPP_Poaceae_ for a similar situation. We used the RPP_Poaceae_ estimates for *Quercus* from the studies by Li et al. (2017) and Zhang et al. (2017) to calculate the mean *Quercus* RPP_Poaceae._ We then assumed that this value was representative for temperate China and therefore valid for the study area of Li et al. (2015). RPP_*Quercus*_ for all other tree taxa in Li et al. (2015) were thus converted to RPP_Poaceae_ using the obtained mean *Quercus* RPP_Poaceae_.

In order to develop synthesized RPP datasets for the study region, we generally used the RPP values considered as the most reliable by the authors (see also discussion below). The final selection of the RPP values followed similar criteria to those of Mazier et al. (2012) for the RPP datasets “standard” 2 and 3 (Std2 and Std3) in Europe. These criteria were based on the recognition that there were too few values available to identify with confidence the factors behind between study differences in RPP values. In this review of Chinese RPPs we adopted the same strategy given that the number of RPP values available was also too low for a more critically informed selection of values. We therefore calculated the mean RPPs for each taxon based on a selection of values following consistent rules. The standard error (SE) of the resultant mean RPPs were estimated using the delta method (Stuart and Ord, 1994).

The selection rules for the synthesized dataset alternative 1 (Alt-1) are comparable to those used for the European dataset Std2:

- when the number of available RPP estimates (N) is 5, the most different one or two estimates were excluded, and the mean RPP was calculated based on the remaining estimates;
- when 5 > N >2, the most different estimate was excluded, and the mean RPP was calculated based on the remaining estimates; for *N* = 4 (*Pinus*) all four values were used to calculate the mean RPP as the two most similar values are from the same forest region (Changbai Mountains).
- If *N* = 2, both values were kept unless one of the values was obviously too large, i.e., larger than the most pollen productive trees; e.g., Caryophyllaceae in Inner Mongolia (Li et al., in preparation) and Shandong (Li et al., 2017).

The rules used for the dataset alternative 2 (Alt-2) are comparable to those used for the European dataset Std3, i.e., we applied the same rules as for Alt-1 but also excluded estimates that were considered as less reliable because of the type of landscape (and related vegetation) used in the original study. For example, we assumed that the high RPP_Poaceae_ values for Cyperaceae and Chenopodiaceae obtained from open vegetation areas (Wang and Herzschuh, 2011) in which these plants were common to be more reliable than the low values from wooded vegetation (Zhang et al., 2017) in which the two taxa were not common and flowering was observed to be poor. Similarly, we retained the RPP_Poaceae_ values for *Juglans* obtained in woodlands (Li et al., 2015) and discarded the value obtained in open land (cultural landscapes of the Shandong province; Li et al., 2017). *Juglans* was mainly cultivated and rarely occurring in the wooded areas, i.e., the pollen-vegetation relationship for *Juglans* was considered as atypical in the Shandong study.

## Results

### ERV analysis methodological factors

Data used and ERV analysis choices made by the authors of the reviewed studies are summarized in Tables S1,S2.

Since all studies used a form of the Prentice-Sugita distance-weighting model which requires values of pollen fall speed for all taxa, Table S3 compares those values. The values vary between studies. The estimates for *Artemisia*, Chenopodiaceae, and Cyperaceae in Li et al. (2017), and for Poaceae in Li et al. (2011), are higher than in the other studies. The FSPs for *Pinus* in Li et al. (2015) and Zhang et al. (2017, Changbai) are higher than those in Zhang et al. (2017, Taiyue) and Li et al. (2017), and the estimate for *Quercus* in Li et al. (2017) is larger than the estimates in all the other studies. However, in spite of these differences between studies, the ranking of FSPs is the same in all studies, i.e., *Pinus* > *Quercus* > Cyperaceae > Poaceae > *Artemisia* > Chenopodiaceae.

The RSAP estimates and likelihood function scores from 10 studies are listed in Table 1. The RSAP was not estimated in the study performed on the eastern Alashan Plateau (Li et al., 2011). The RSAP values differ between studies but they are generally large (1,000–2,700 m) except in the study of Li et al. (2017) (145 m) and in the studies of He et al. (2016) (8–20 m) and Li et al. (in preparation; 25 m). The RSAP estimates obtained in the open landscapes of the Tibetan Plateau and Inner Mongolia, and in the forests of the Changbai and Taiyue mountains, have the same order of magnitude (around 2,000 m), with both the largest and smallest estimates (2,700 m; Ge et al., 2015 and 1,000 m; Xu et al., 2014) from studies in central Inner Mongolia.

**Table 1:** Likelihood function score at the distance corresponding to the relevant source area of pollen (RSAP) as identified by the authors of the eleven studies reviewed in this study.

**References**	**Submodel**	**RSAP**	**Likelihood function score**
Li et al., 2011	1 and 2	NA	NA
Wang and Herzschuh, 2011	2	2200	6190-6210
Wu et al., 2013	1	1000	16271.4
	2	1000	15939.5
Xu et al., 2014	2	1000	NA
Li et al., 2015	1	2000	11030
	2	2000	11100
	3	2500	10900
Ge et al., 2015	1	around 2000	around 54800
	2	2700	54396
	3	2100	54255
Li et al., 2017	1	173	62528.1
	2	92	62113.9
	3	145	63058
Zhang et al., 2017 (Changbai)	3	1000	41048.2
Zhang et al., 2017 (Taiyue)	3	2000	25345
He et al., 2016 (Hulunbeier)	2 and 3	20	9900
He et al., 2016 (Xilinhaote)	1 and 3	9	4525
He et al., 2016 (Sunitezuoqi)	1, 2 and 3	8	9825-9850
Han et al., 2017	1	1600	6000
Li et al., in preparation	1	25	20955

*Note that the likelihood function scores and the RSAP values cannot be compared between studies given that they were not calculated with comparable pollen-vegetation datasets. See text for more explanations*.

### Comparison of relative pollen productivity (RPP) estimates between studies and proposed standard datasets of RPP estimates

Table 2 summarizes RPP_Poaceae_ (hereafter RPP) estimates for 38 taxa (excluding the reference taxon Poaceae) from the 11 studies. These estimates cover 11 tree taxa, four shrub taxa and 24 herb taxa (Table 2). For 23 of these 38 taxa, there is one RPP estimate available so far, comprising four tree taxa (Cupressaceae, *Castanea, Picea*, and *Robinia/Sophora*), four shrub taxa (Elaeagnaceae, *Vitex negundo, Nitraria*, and *Hippophae*), and 15 herb taxa (Convolvulaceae, *Cannabis*/*Humulus*, Liliaceae, *Potentilla* type, *Sanguisorba* type, Ranunculaceae, *Thalictrum, Ephedra, Galium*, Compositae SF Cichorioidae, *Aster/Anthemis* type, *Stellera*, Iridaceae, Lamiaceae, and *Mentha* type (*Thymus* in the original publication)). For the remaining 15 taxa, i.e., *Pinus, Juglans, Betula, Ulmus, Larix, Quercus, Fraxinus, Tilia*, Amaranth./Chenop., Caryophyllaceae, Brassicaceae, *Artemisia*, Fabaceae, Compositae, Cyperaceae, the values obtained vary between sites. The tree taxa *Pinus* and *Quercus* have high and relatively consistent RPP values in the cultural landscape of the Shandong province and the wooded landscapes of the Changbai and Taiyue mountains, although the RPP of *Pinus* is significantly higher in the Taiyue Mountain, and one of the values obtained in the Changbai Mountain for *Quercus* is very low. The *Larix* values from the Changbai and Taiyue Mountains vary between sites. Cupressaceae, *Robinia/Sophora* and *Tilia* have the lowest RPP estimates among tree taxa. The largest differences in RPP values between studies are found for *Ulmus* and *Juglans*. The RPP estimate for *Ulmus* in the Changbai Mountains (Li et al., 2015) is much higher than the value from the Shandong province (Li et al., 2017), and the RPP estimates for *Juglans* in the Taiyue (Zhang et al., 2017) and Changbai Mountains (Li et al., 2015) are much higher than those from the Shandong province (Li et al., 2017) and elsewhere in the Changbai Mountains (Zhang et al., 2017).

**Table 2:** Estimates of relative pollen productivity (RPP) from the eleven studies reviewed in this paper.

**Studies**	**Li et al., 2011 (1)**	**Wu et al., 2013 (2)**	**Xu et al., 2014**	**Wang and Herzschuh**, **2011**		**Li et al.**, **2015** **(2)**			**Ge et al., 2015**	**He et al., 2016, Hulunbeier (3)**	**He et al., 2016, Xilinhaote (3)**	**He et al., 2016, Sunitezuoq (3)**	**Han et al., 2017**	**Li et al.**, **2017**			**Zhang et al., 2017 Changbai (4)**	**Zhang et al., 2017 Taiyue (4)**	**Li et al., in preparation**
**Taxa**	**model1.soil**	**model 1**	**model 1**	**model 1**	**model 2**	**model 1**	**model 2**	**model 3**	**model 3**	**model 3**	**model 3**	**model 1**	**model 1**	**model 1**	**model 2**	**model 3**	**model 3**	**model 3**	**model 1**
Pinus^*^						6.53 ± 0.36	26.91 ± 0.36	16.13 ± 0.52^*^					20.07 ± 6.96	7.82 ± 0.24	12.12 ± 0.1	8.96 ± 0.23^*^	18.82 ± 0.54^*^	29.55 ± 1.77^*^	
Cupressaceae^*^														0.51 ± 0.06	0.45 ± 0.04	1.11 ± 0.09^*^			
Quercus^*^		100 ± 0				5.19 ± 0.09	5.19 ± 0.09	5.19 ± 0.09^*^					58.05 ± 9.54	4.25 ± 0.2	5.65 ± 0.11	4.89 ± 0.16^*^	1.75 ± 0.31	5.48 ± 0.11^*^	
Juglans^*^						8.66 ± 1.04	10.37 ± 1.92	10.06 ± 0.47						0.96 ± 0.11	0.94 ± 0.08	0.3 ± 0.05	1.69 ± 0.24	7.69 ± 0.49^*^	
Castanea^*^														4.63 ± 0.31	6 ± 0.38	11.49 ± 0.49^*^			
Betula^*^		16 ± 1				21.67 ± 1.81	19.7 ± 1.81	26.13 ± 0.78^*^					1.16 ± 0.43				11.67 ± 0.22^*^	13.16 ± 0.08^*^	
Fraxinus^*^						1.92 ± 0.47	6.01 ± 1.19	3.94 ± 0.73^*^									0.21 ± 0.06		
Ulmus^*^			12.41 ± 1.14			6.74 ± 0.99	10.78 ± 1.97	7.26 ± 1.81^*^					9.44 ± 3.50	1.28 ± 0.28	0.42 ± 0.14	1 ± 0.31^*^	0 ± 0.17		
Larix^*^						1.45 ± 0.16	3.01 ± 0.36	1.56 ± 0.21^*^									4.41 ± 0.15^*^	3.87 ± 0.6^*^	
Robinia/Sophora^*^														0.69 ± 0.03	0.85 ± 0.02	0.78 ± 0.03^*^			
*Picea*		296 ± 44																	
Poaceae^*^	1	1	1	1	1^*^			1^*^	1	1	1	1	1	1	1	1	1	1	1
*Vitex negundo*														0.12 ± 0.02	0.07 ± 0.02	0 ± 0.02			
**Sanguisorba type**																			24.07 ± 3.50
Potentilla type^*^			1.12 ± 0.15						0.22 ± 0.09^*^										
Polygonaceae																			
Ranunculaceae^*^																		7.86 ± 2.65^*^	
Artemisia^*^	226.43	908 ± 41	11.05 ± 0.26	2.08 ± 0.43	3.27 ± 0.63				19.33 ± 0.41^*^	2.44	100.00	0.47	1.29 ± 0.27	23.27 ± 0.72	15.51 ± 0.27	24.7 ± 0.36^*^	21.53 ± 2.16^*^	0.01 ± 1.6	19.03 ± 0.27^*^
*Nitraria*	20																		
Cyperaceae^*^		1 ± 0	0.86 ± 0.08	1.04 ± 0.01	0.66 ± 0.03^*^				8.9 ± 0.33	2.66	0.13	0.19	0.01 ± 0.01	0.16 ± 0.08	0.5 ± 0.08	0.21 ± 0.07^*^	0.05 ± 0.07	0.03 ± 0.07	
Amaranth./Chenop.^*^	71.43		5.95 ± 0.78	5.44 ± 1.23	5.34 ± 1.08^*^				21.01 ± 2.47	10.88	205.00	16.85	50.49 ± 3.56	0.7 ± 0.11	0.29 ± 0.11	0.18 ± 0.16			3.57 ± 0.81^*^
Brassicaceae^*^			7.56 ± 0.34											0.14 ± 0.06	0 ± 0.03	0.89 ± 0.18^*^			
*Thalictrum*			2.83 ± 0.40																
*Iridaceae*			0.01 ± 0.25																
Compositae^*^			0.18 ± 0.15						7.73 ± 0.54^*^	0.73	472.00	3.13	0.19 ± 0.20			1.06 ± 0.21^*^			
Aster/Anthemistype^*^														1.35 ± 0.08	0.83 ± 0.07	1.26 ± 0.4^*^			
Compositae SF. Cichorioideae^*^														1.49 ± 0.1	0.53 ± 0.05	0.86 ± 0.11^*^			
*Ephedra*			1.25 ± 0.18																
*Convolvulaceae*									0.18 ± 0.03^*^										
Fabaceae^*^									0.2 ± 0.1^*^							0.78 ± 0.03^*^			
Lamiaceae^*^									0.2 ± 0.13^*^										
**Mentha type(Thymus)**																			2.27 ± 0.35^*^
*Liliaceae*									1.49 ± 0.11^*^										
Caryophyllaceae														0.66 ± 0.06	0.47 ± 0.08	0.87 ± 0.14			78.20 ± 5.85
Galium type^*^														0.32 ± 0.13	0.45 ± 0.16	1.23 ± 0.36^*^			
*Stellera*																			33.05 ± 3.78
Cannabis/Humulus^*^														14.59 ± 0.68	5.2 ± 0.25	16.43 ± 1^*^			
Elaeagnaceae^*^																		8.88 ± 1.3^*^	
*Hippophae*		167 ± 19																	

**Figure 4:**
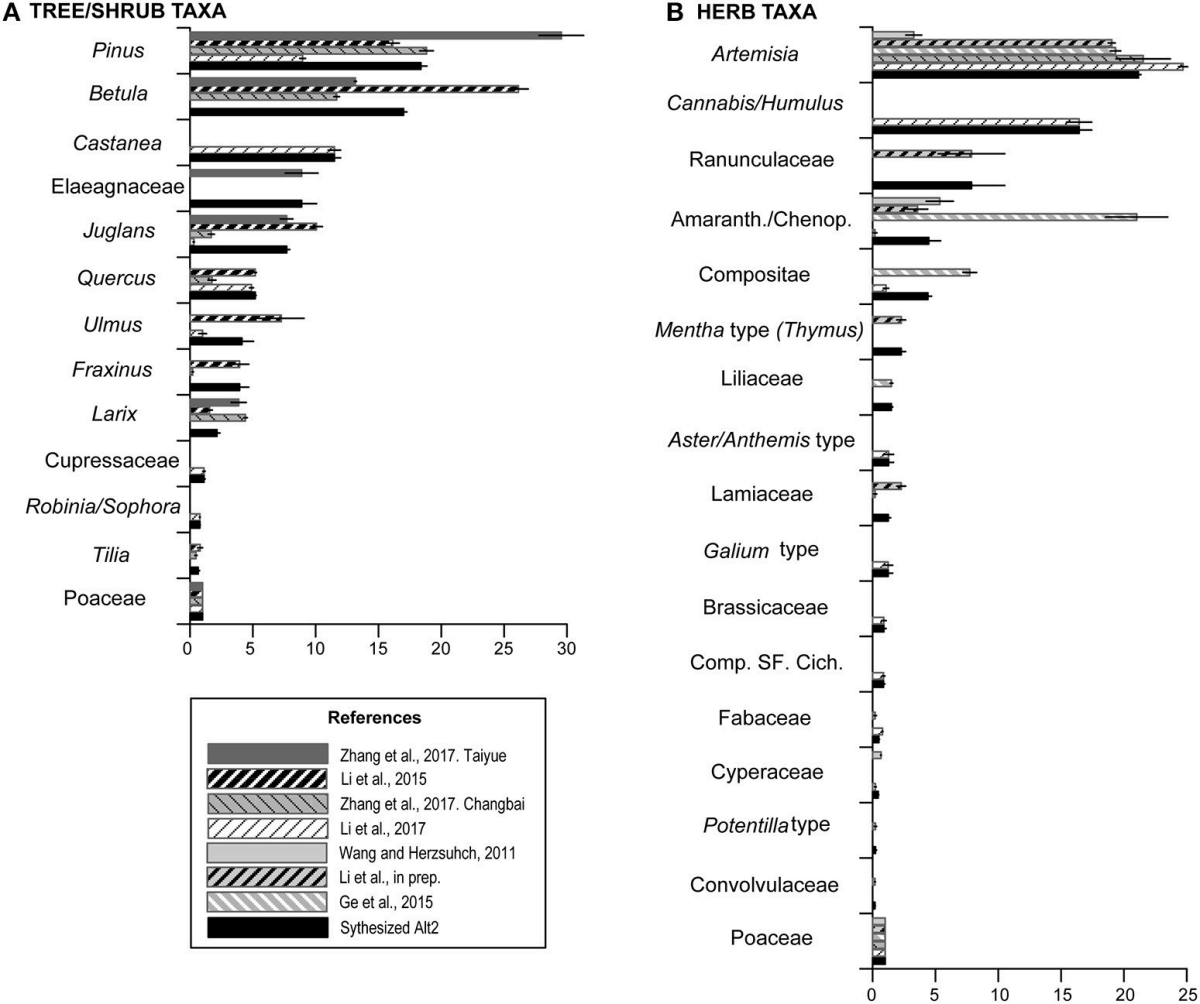
Relative pollen productivity (RPP) estimates of tree, shrub, and herb taxa related to Poaceae set to 1 from seven selected study regions, and mean of selected RPP estimates according to the rules applied for the synthesized RPP dataset Alt-2. **(A)** RPPs for tree/shrub taxa. **(B)** RPPs for herb taxa. See text for more explanations.

All RPP values are related to Poaceae (RPP = 1). For the six selected studies (seven study regions) used for the synthesized RPP datasets (Figure 4; Table 3), we present all the RPP values from the ERV model selected as the most reliable one by the authors, while only one model results are presented for the studies from which RPP values were not selected for the synthesized RPP datasets. The studies with RPP values selected for Alt-1 and Alt-2 are emphasized in bold. The taxa and their RPP values included in the synthesized RPP data set Alt-1 are underlined and those included in Alt-2 are marked with

* *(see Table 2). The study for which the RPPs related to Chenopodiaceae were converted to RPPs related to Poaceae is indicated by (1). The studies for which the RPPs related to Quercus were converted to RPPs related to Poaceae is indicated by (2). The studies for which the RPPs related to Artemisia were converted to RPPs related to Poaceae are indicated by (3). The studies for which the ERV analysis was redone using Poaceae as reference taxon instead of Quercus are indicated with (4)*.

Among the herbs, *Artemisia* and Chenopodiaceae have the highest RPP estimates. The RPP values are particularly high in the study from the Alashan Plateau (*Artemisia*: 226) and Chenopodiaceae: 71). Values for *Artemisia* from the Tibetan Plateau (3.27) and the Changbai mountains (5.34) are relatively low, and those from Inner Mongolia (four studies) lie between 19 and 25. Amaranth./Chenop. has relatively low values in the Shandong study and one of the Changbai Mountain studies (Zhang et al., 2017), and middle or high values in the Inner Mongolia and Tibetan Plateau studies. The values from the Alashan Plateau for both *Artemisia* and Amaranth./Chenop. are markedly different from the values of the other studies. We therefore chose to exclude all RPP values from that study for the calculation of the mean RPPs. Cyperaceae has either low (Tibetan Plateau, 0.66; Shandong, 0.21), or very high (Inner Mongolia, 8.9) RPP estimates relative to all available values.

We excluded the RPP values of four additional studies, those from central Inner Mongolia (Xu et al., 2014), the Xinglong Mountains (Wu et al., 2013), and the forest-steppe ecotone in the Xilinguole and Hulunbeier regions (Han et al., 2017) and the forest-steppe ecotone in Hulunbeier, steppe in Xinlinhaote, and desert in Sunitezuoqi (He et al., 2016) for the following reasons:

- In the Xu et al. (2014) study, the likelihood function scores increase with distance where they theoretically should decrease (Sugita, 1994). The reason for this is not known for that particular study. However, such results are generally due to violation of one or several assumptions of the ERV-model (reviewed under Methods above), e.g., sites not randomly distributed in the landscape, sampling across different vegetation regions, or poor selection of taxa for inclusion in the data analysis (Li et al. unpublished results).
- In the case of the Wu et al. (2013) study, we consider that the results are not comparable with those of the other studies because the pollen samples (moss polsters) were collected within a much larger area (10 m × 10 m) than in the other studies (generally within a 0.5 m radius area), therefore effectively a much larger sampling basin was used. Although this should not influence the results if it is taken into account in the analysis, it is not possible to tell whether this did occur from the publication.
- Han et al. (2017) obtained RPP_Poaceae_ estimates for eight plant taxa. These RPPs show large discrepancies from most of the values included in our synthesis. Except for *Pinus* (20.07), the RPP estimates are either much higher than the other values in Table 3 (Figure 4), i.e., *Quercus* (58.05), *Ulmus* (9.44), and Amaranth./Chenop. (50.49), or much lower, i.e., *Betula* (1.16) and *Artemisia* (1.29). The values for Compositae (0.19 ± 0.20) and Cyperaceae (0.01 ± 0.01) have standard errors equal or larger than the RPP, which implies that the estimate is equal to zero. These large discrepancies are most likely due to the heterogeneous vegetation within the study area; the region chosen cover an ecotone with a succession of very different vegetation types (see methods and discussion sections, and Figure 3). The heterogeneity of the vegetation within the study region might also explain the atypical likelihood function score plots. The authors also estimated the relevant source area of pollen (RSAP) for subsets of lakes located in sub regions of the wider study area, therefore characterized by more homogenous vegetation. The groups of lakes were also more homogenous in terms of the lake's size (large, medium or small). In those cases, the likelihood scores decrease with distance as expected. No RPP values were presented for these sub-sets.
- The RPP values from the study of He et al. (2016) were based on pollen collected from pollen traps which behave differently than pollen assemblages collected from soils, moss polsters or lake sediments (e.g., Lisitsyna et al., 2012).

**Table 3:** Synthesized RPP datasets, Alt-1, and Alt-2.

**Taxa**	**Alt-1**	**Alt-1.SE**	**Alt-2**	**Alt-2.SE**	**Europe**
Amaranth./Chenop.	3.03	0.45	4.46	0.68	4.28 ± 0.27
*Artemisia*	17.57	0.47	21.15	0.56	3.48 ± 0.20
*Aster/Anthemis* type	1.26	0.4	1.26	0.4	
*Betula*	16.99	0.27	12.42	0.12	3.09 ± 0.27
Brassicaceae	0.89	0.18	0.89	0.18	
*Cannabis/Humulus*	16.43	1	16.43	1	
*Castanea*	11.49	0.49	11.49	0.49	
Comp. SF. Cich.	0.86	0.11	0.86	0.11	0.16 ± 0.02
Compositae^*^	4.4	0.29	4.4	0.29	
Convolvulaceae	0.18	0.03	0.18	0.03	
Cupressaceae	1.11	0.09	1.11	0.09	2.07 ± 0.04 *Juniperus*
Cyperaceae	0.44	0.04	0.44	0.04	0.87 ± 0.06
Elaeagnaceae	8.88	1.3	8.88	1.3	
Fabaceae^*^	0.49	0.05	0.49	0.05	
*Fraxinus*	2.08	0.37	3.94	0.73	1.03 ± 0.11
*Galium* type	1.23	0.36	1.23	0.36	2.61 ± 0.23 Rubiaceae
*Juglans*	3.23	0.18	7.69	0.24	2.35 ± 0.11 *Fagus*
Lamiaceae^*^	1.24	0.19	1.24	0.19	
*Larix*	3.28	0.22	2.14	0.24	
Liliaceae	1.49	0.11	1.49	0.11	
*Mentha* type *(Thymus)*	2.27	0.35	2.27	0.35	
*Pinus*	18.37	0.48	18.37	0.48	6.38 ± 0.45
Poaceae	1	0	1	0	1
*Potentilla* type	0.22	0.09	0.22	0.09	1.19 ± 0.14
*Quercus*	4.33	0.09	5.19	0.07	5.83 ± 0.15
Ranunculaceae	7.77	1.56	7.77	1.56	1.96 ± 0.36 *Ranunculus acris-t*
*Robinia/Sophora*	0.78	0.03	0.78	0.03	
*Sanguisorba* type	24.07	3.5	*NA*	*NA*	
*Stellera*	33	3.78	*NA*	*NA*	
*Tilia*	0.65	0.11	0.65	0.11	0.80 ± 0.03
*Ulmus*	4.13	0.92	4.13	0.92	1.27 ± 0.05

The standard errors for the mean RPP values in the two alternatives are calculated using the delta method (Stuart and Ord, 1994). The RPP values for taxa marked with

* *are mean values combined from RPPs for individual taxa of lower hierarchical rank. The mean RPPs from the standard dataset for Europe are shown for comparison (from “standard 2” in Mazier et al., 2012, except for Amaranth./Chenop. that is from Abraham and Kosakova, 2012). See caption of Table 2 and main text for more explanations*.

In the Alt-1 dataset, tree taxa have generally higher RPP estimates than herbs. The RPP ranking of tree taxa is as follows: *Pinus* > *Betula* > *Castanea* > *Quercus* > *Ulmus* > *Larix* > *Juglans* > *Fraxinus* > Cupressaceae. Only three herb taxa have values comparable to trees. *Artemisia* and *Cannabis/Humulus* have values similar to *Pinus*, and the value for Amaranth/Chenop. lies between *Quercus* and *Ulmus*. The rank order of the remaining taxa is Liliaceae > Lamiaceae ≈ *Galium* type > [Poaceae = 1] > Fabaceae > Cyperaceae > *Potentilla* type and Convolvulaceae. The Alt-2 dataset is very similar to Alt-1 in terms of values and ranking. Within trees, *Betula* (16.99) has a lower mean RPP value than in Alt-1, but is still ranked between *Pinus* and *Castanea*, while *Juglans* (7.69) has a higher mean RPP value than *Quercus* and *Ulmus*. Within herbs, *Artemisia* (21.15) and Amaranth/Chenop. (4.46) have higher mean RPP values in Alt-2 than in Alt-1, but they keep the same ranking.

## Discussion

### Possible causes of between-study differences in RSAP and RPP estimates

RPP studies in Europe suggest that the two major groups of factors underlying between-study discrepancies in RPP estimates arise from (i) methodological issues, e.g., between-study differences in site selection, pollen, and vegetation data collection protocol, choice of reference taxon, method used to estimate the RSAP, and ii) environmental factors, i.e., differences between pollen productivity for the taxa of interest related to differences in climate, landscape management, and structure, vegetation spatial structure, and/or species composition (e.g., Broström et al., 2008; Bunting et al., 2013). In this section, we consider the extent to which the same two groups of possible factors may underlie between-study and between-region differences in RPP values. We excluded five studies from the synthesized RPP data sets Alt-1 and Alt-2 due to methodological issues. These studies also exhibit RPP values that differ significantly from the values obtained in the six retained studies (Table 2). This suggests that methodological factors likely represent the most important reason behind differences in RPP values between studies.

The remainder of this section focuses on the six “reliable” studies, i.e., studies performed with comparable methods. Table 4 summarizes the inferred effects of aspects of each of the two groups of factors on RPP estimates for different pollen taxa. Where the differences in RPPs between studies can be ascribed mainly to differences in one factor, we considered that to be a “likely effect.” If the factor's characteristics differ between the studies, but the RPP values are very similar, we infer that the factor has no effect on the RPP values and it is indicated as “no effect”. If the effect of a factor cannot be inferred clearly from the available RPP values, we consider that the factor may have an effect and it is indicated as a “possible effect.”

**Table 4:** Summary of methodological and environmental factors and their probable effect on RPP values.

	**Methodological factors**			**Enviromental factors**		
**Taxa\Factors**	**Sites selection**	**Vegetation collection**	**Other factors**	**Different species**	**Vegetation/landscape characteristics**	**Climate**
*Pinus*	⊙	⊙	⊙	⊙	⦿	⦿
*Quercus*	⊙	⊙	⊙	⊙	⊙	⊙
*Juglans*	⊙	⊙	⊙	⊙	⦿	⊖
*Betula*	⊙	⦿	⊙	⊖	⊖	⊖
*Fraxinus*	⊙	⦿	⊙	⊖	⊖	⊖
*Ulmus*	⊖	⊙	⊙	⦿	⦿	⊙
*Larix*	⊙	⦿	⊙	⊖	⊖	⊖
*Artemisia*	⊙	⊙	⊙	⊖	⊖	⊖
Poaceae (reference)	⊙	⊙	⊙	⊙	⊙	⊙
Cyperaceae	⊙	⦿	⊙	⊖	⊙	⊖
Amaranth./Chenop.	⦿	⦿	⊙	⦿	⦿	⊖
Compositae	⦿	⦿	⊙	⦿	⦿	⊙
Fabaceae	⦿	⦿	⊙	⦿	⦿	⦿
Lamiaceae	⊖	⦿	⊙	⊙	⊙	⊖
⦿ Likely effect	⊙ Possible effect	⊖No effect				

*If the differences in RPPs between studies can be ascribed mainly to one factor, it is indicated in the table as “likely effect.” If the effect of a factor cannot be inferred clearly from the available RPP values, we consider that the factor may have an effect and it is indicated as “possible effect.” If the factor's characteristics differ between the studies, but the RPP values are very similar, we infer that the factor has no effect on the RPP values and it is indicated as “no effect.” “Other factors” are other methodological factors such as types of pollen sample (moss polster, soil, lake sediment), pollen site distribution and selection of plant taxa for RPP calculation*.

#### Methodological factors

The study landscape should be selected based on the vegetation types/taxa of interest, but not the sampling sites. A suitable region needs to have a homogenous mosaic of vegetation types. Within such a region, the differences between the methods used to locate the sample points and assemble the vegetation dataset probably have the largest influence on the RSAP and RPP values. True random distribution of sites was used only in two studies (Taiyue Mountain, Zhang et al., 2017; Shandong province, Li et al., 2017). In the other studies, sites were selected based on the vegetation types/taxa of interest occurring in a region and/or the location close to roads.

Methods used for collection of vegetation data beyond 100 m also differ between studies in terms of source data (vegetation maps or satellite images) and resolution of the spatial representation of the vegetation units. The distances used for different details of vegetation recording also vary between studies, for instance up to 10 m or 20 m for the detailed surveys, and up to 1,000 m, 1,500 m, or 5,000 m for extraction of vegetation data beyond 100 m. After site distribution, between-study differences in the size and distribution of vegetation units in the vegetation surveys and maps will influence RSAP significantly. Simulation studies using hypothetical landscape/vegetation structures or mimicking actual vegetation have shown that the smaller the patches and the more homogenous the patches and taxa distribution, the smaller the RSAP (e.g., Bunting et al., 2004; Bunting and Gaillard in Gaillard et al., 2008; Hellman et al., 2009a,b). The methods used for collection of vegetation data (taxa composition) within 10 m and between 10 and 100 m (quadrats in all studies) imply that vegetation “structure” in the final vegetation input data for the ERV model is expressed as a homogenous distribution of taxa with various cover within rings of increasing surface around the pollen sample. The taxa distribution depends on the mean taxa composition obtained from the surveyed squares within the distances of the successive rings. In a mosaic vegetation/landscape, one can assume that the more detailed the differentiation and recording of patches whether in the field, from maps, or satellite images, the larger the change in taxa composition between rings may become. A low-resolution vegetation map (1:1,000,000) was used to extract vegetation data beyond 100 m in Inner Mongolia (Ge et al., 2015; Li et al., manuscript in preparation), the Taiyue Moutains (Zhang et al., 2017) and one of the studies in the Changbai Mountains (Zhang et al., 2017), while a more detailed forest map was used in the other Changbai Mountains' study (Li et al., 2015). Very detailed land-use maps were used in the Shandong study (Li et al., 2017). Hellman et al. (2009a) showed that the larger the community patches, the larger the RSAP. Therefore, because low-resolution vegetation maps may imply larger community patches than high-resolution maps, the resolution of maps may impact on the RSAP, and in turn on the RPP results. Moreover, the somewhat “noisy” behavior of the log likelihood values in the study of Li et al. (2017) might be due to the very detailed recording of vegetation patches. Mazier et al. (2008) obtained similar curves in the cultural landscape of the Jura Mountains in Europe using 1 m increments for extraction of vegetation data. Low-resolution extraction of vegetation data (e.g., 5–10 m) will produce a less “noisy” curve of log likelihood or likelihood function scores. As vegetation survey and mapping cannot practically be precise at the 1 m × 1 m scale, it might be more relevant to work with 5 to 10 m resolution.

The fall speed of pollen (FSP) used for the taxa involved in the analysis (Table S3) will also have an effect on the RSAP distance and RPP estimates obtained. FSP is usually calculated using Stoke's law (Gregory, 1973) and measurements of the size of the pollen grains. The values of FSP may differ between studies due to (i) the measurements of pollen grains, i.e., the selected species for which measurements are performed for a particular taxon, or the preparation and mounting methods in the reference collection used, and (ii) the method of calculation including (or not including) adjustment by a shape factor and using (or not using) a lower density for saccate pollen grains (0.5 instead of 1). Adjustment by shape factor and low density for saccate grains were applied in the studies by Li et al. (2017), Zhang et al. (2017) and Li et al. (manuscript in preparation), which explains the differences of FSP values between these studies and the other eight studies reviewed in this paper. Simulation studies using the ERV model showed that changing the FSP values of plant taxa notably affects pollen loading in lakes and bogs (Bunting et al., 2004). Therefore, differences in FSP values will affect RSAP and RPP values obtained from pollen and vegetation data and the application of ERV models. Therefore, a synthesis of available values for fall speed of pollen might be valuable for future RPP studies.

The type of pollen trap sampled may also have an effect on the RPP values because pollen taphonomy and preservation will differ between sample types (surface soil, lake sediment, moss polster, or artificial pollen trap). It is known that there is a high annual variability in artificial pollen trap data (Hicks et al., 2001). Moss polsters also represent a short time of pollen deposition [generally 1–2 (3) years according to European studies; e.g., Cundill, 1991; Räsänen et al., 2004]. Lake-surface sediments will include a variable number of years depending on the accumulation rate of the sediment and the thickness of the sediment sample. Pollen assemblages in soil samples are often very different from pollen assemblages in pollen traps and moss polsters due to biases from selective preservation of pollen grains in soil and downwards water movement carrying part of the pollen grains to lower soil layers (e.g., Xu et al., 2016; Zhang et al., 2016). For instance, the RPP estimates of *Artemisia* using pollen data from lake surface sediments (Wang and Herzschuh, 2011) are much lower than those obtained from soil and moss samples, which may be due to processes in lakes' water body that bias pollen deposited in surface sediments.

Finally, the choice of ERV submodel and method to distance weight vegetation data do influence significantly the values of RSAP and RPP. Theoretically, the taxon-specific method should be the soundest method to distance weight vegetation, given the pollen dispersal model used is appropriate. The most commonly used dispersal model is a Gaussian-plume (GP) model, which is the model used in the ERV model of Prentice (Parsons and Prentice, 1981; Prentice and Parsons, 1983). This is also the model used in all studies reviewed here. More recently, Theuerkauf et al. (2012) used a Lagrangian stochastic (LS) model. The authors argue that the LS model is more appropriate than the GP model for heavy pollen grains such as *Fagus* and *Picea*. This suggests that RPP studies should in the future use both dispersal models and evaluate the results by testing the obtained RPP values using modern pollen-vegetation datasets.

Among the three ERV submodels, the ERV submodel 3 should be the most adequate model to use when absolute vegetation data (in m^2^/m^2^) are available. Following Sugita (1994), the combination of ERV submodel and distance-weighting method giving the lowest likelihood function scores (highest log likelihood) is usually selected as the combination providing the best RPP values (e.g., Mazier et al., 2008; Wang and Herzschuh, 2011). These scores indicate that the correction factors estimated (RPP and background term) produce the best fit with the actual data. Among the 11 studies reviewed, five studies (Ge et al., 2015; Li et al., 2015, 2017; Zhang et al., 2017; Li et al., manuscript in preparation) used absolute vegetation data in m^2^/m^2^. Four of these found that sub-model 3 produced the best fit, whilst Li et al. (manuscript in preparation) found that sub-model 1 produced the lowest log-likelihood scores. Submodel 3 was not used in the other six studies, because absolute vegetation data were not available. Moreover, the authors did not specify which submodel was considered to provide the best results.

Despite the variation in methods between studies, the estimates of RSAP summarized in Table 1 are all similarly large (1,000–2,500 m with ERV submodel 3) except for the Shandong study (145 m). This indicates that the most probable factor behind the large difference between that study and all others is the very complex landscape/vegetation mosaic of Shandong's cultural landscapes compared to the forest landscapes and the semi-natural, extensively managed open meadows and steppes of the other study areas. The patch size and distribution of vegetation units recorded in field within 100 m of the pollen samples, and on the vegetation maps beyond 100 m, are smaller and more homogenous than those in the other landscapes studied in China (Figure 2; vegetation maps in Li et al., 2015).

#### Environmental factors

The three major environmental factors that may affect the RPP_Poaceae_ values presented in Tables 2, 3 are the plant species included in the pollen taxa, climate of the study region, and vegetation/landscape structure (Table 4).

Taxonomic issues seem to play a major role in the observed RPP differences for some taxa. For example, the genus *Artemisia* is represented mainly by the species *A. desertorum, A. frigida*, and *A. canacetifolia* in Inner Mongolia, by *A. annua, A. sacrorum* and *A. gmelinii* in the cultural landscapes of Shandong, and by *A. argyi* on the Tibetan Plateau. However, the RPP values of *Artemisia* from Inner Mongolia (Ge et al., 2015; Li et al., manuscript in preparation), the Changbai Mountains (Zhang et al., 2017) and Shandong province (Li et al., 2017) are comparable. The low values from the Tibetan Plateau (Wang and Herzschuh, 2011) are probably due to other factors, such as the pollen sample type (lake sediments). The taxon Amaranth./Chenop. is represented mainly by *Portulaca oleracea, Achyranthes bidentate, Chenopodium album, Salsola collina*, and *S. komarovii* in Shandong, *Haloxylon ammodendrom* in Inner Mongolia and by *Chenopodium hybridum* on the Tibetan Plateau. The *Chenopodium* species tend to have larger pollen grains than the other species of Amaranth./Chenop. (e.g., Beug, 2004; Tang et al., 2016). Although differences in RPPs of Amaranth./Chenop. between studies occur, it is again more probable that they are due to the vegetation structure and land-use management (different in alpine meadows and steppes compared to agricultural land) and the total cover of the pollen taxon in the vegetation (very low in the Shandong agricultural land compared to the other areas). The pollen type *Pinus* is mainly produced by *P. koraiensis* in the Changbai Mountains, *P. tabulaeformis* in the Taiyue Mountains, and by *P. tabulaeformis* and *P. thunbergii* in Shandong. Although the species are different, the RPP values obtained from the two studies from the Changbai Mountains (Li et al., 2015; Zhang et al., 2017) are comparable, while the RPP value from the Taiyue Mountains (Zhang et al., 2017) is significantly larger and that from Shandong (Li et al., 2017) much lower than the Changbai Mountains' values. The major difference between the Taiyue Mountains and Shandong is the landscape/vegetation type and structure, and methods of vegetation data-collection beyond 100 m, while the major difference between those two regions and the Changbai Mountains is climate. Therefore, the reasons behind the between study differences in RPPs might be the vegetation structure or/and differences in climate, and cannot be disentangled further.

Whether the differences in climate between the study regions have a major effect on the RPP estimates is still an open question. Whilst there are strong climatological differences between the steppe communities in northern China and the cultural landscape of Shandong in central-eastern China, the taxon *Artemisia* in those two regions produce comparable RPP_Poaceae_ estimates. See also the discussion on *Pinus* RPPs above.

### Comparison of RPP estimates between temperate China and Europe

**Figure 5:**
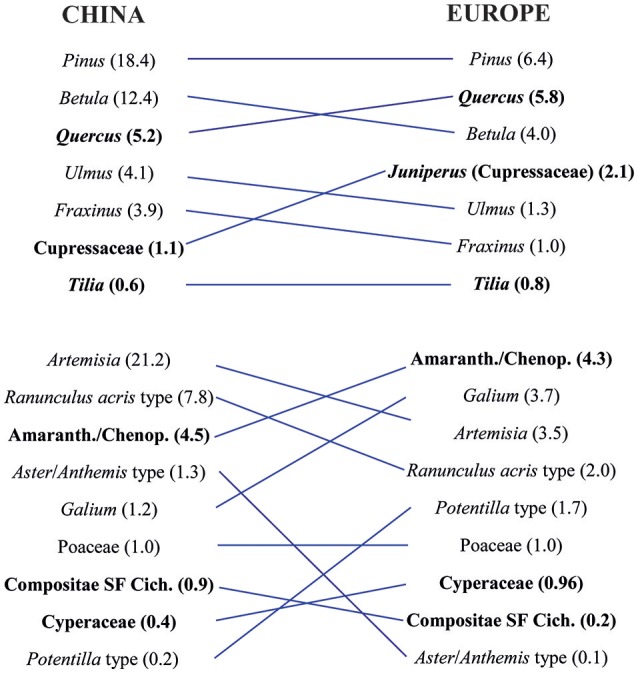
Ranking of Chinese (this review, synthesized dataset Alt-2) and European [Mazier et al., 2012 except for Amaranth./Chenop. from Abraham and Kozáková (2012)] relative pollen productivity (RPP) estimates of tree and herb taxa related to Poaceae set to 1. To facilitate comparison of the RPP values for each taxon and the ranking of taxa between China and Europe, the taxa were linked by blue lines.

A comparison of the RPP estimates obtained so far in China with those from Europe for 15 taxa (Table 3) shows a number of discrepancies and similarities that are interesting. The estimates are generally higher in China than in Europe except for Cupressaceae and *Potentilla* type. However, the position in ranking of taxa relative to Poaceae (i.e., whether RPP_Poaceae_ is >1 or < 1) are very similar in China and Europe although the actual values can differ by as much as an order of magnitude (Figure 5). Exceptions are the trees *Betula* and *Cupressaceae*, and the herbs *Artemisia, Aster/Anthemis* type and *Potentilla* type for which ranking clearly differs between the two regions. The RPP estimates of *Quercus* are comparable in Europe and China in spite of the different species involved. The RPP estimates of *Artemisia* in Europe are six times lower than in China most probably due to the species involved and the different vegetation types dominated by this genus. The RPP for Amaranth./Chenop. on the Tibetan Plateau (Wang and Herzschuh, 2011) is comparable to the RPP value of Amaranth./Chenop. in Europe (Abraham and Kosakova, 2012), although the RPP estimate from Inner Mongolia is four times higher (Ge et al., 2015).

It is clear that more studies are needed to test these proposed sets of values and to provide new RPP values for the taxa discussed in this paper, in particular for those with a single estimate so far. Moreover, we still lack RPP estimates for other major taxa in temperate China, e.g., *Acer, Alnus, Carpinus, Carya, Celtis, Picea, Abies, Corylus*, Apiaceae, Euphorbiaceae, *Ephedra, Plantago, Salix, Fagus, Thalictrum*, and Cerealia type, all of which are common in fossil pollen records of temperate China (Cao et al., 2013).

## Conclusions

Relative pollen productivity (RPP) of plant taxa is one of the key parameters required for quantitative reconstruction of vegetation abundance using the Landscape Reconstruction Algorithm (LRA; Sugita, 2007a,b) or the Multiple Scenario Approach (MSA; Bunting and Middleton, 2009). One important assumption in these models is that the relative pollen productivity of plants is constant in space and time. However, RPP studies in Europe (Broström et al., 2008 and Mazier et al., 2012) and China (this study) show that the RPP estimates of major taxa can differ significantly between studies. Most of the discrepancies between estimates can be explained by either methodological or environmental differences between the study regions. However, the results so far suggest that methodological issues and differences in vegetation/landscape structure are the major factors behind between-studies differences in obtained RPPs. The latter implies that environmental factors such as climate in the study regions and species involved in the studied taxa might be less important and, in many cases, likely do not influence RPP values significantly. Nonetheless, this is not true for all taxa studied so far, and we still need a much larger number of RPP values to reach more reliable conclusion on this issue.

Given the many differences between the studies discussed above, we applied similar rules as Mazier et al. (2012) to establish two alternative synthesized RPP datasets for temperate China, Alt-1, and Alt-2 (see methods). The RPP dataset Alt-2 (Table 3; Figure 4) is recommended for applications of the REVEALS and LOVE models (Sugita, 2007a,b) in temperate China until more RPP estimates are available for these plant taxa and further comparison between values and evaluation can be achieved (see first section of the discussion). The proposed synthesized RPP dataset supports two lines of future research. First, the values can be tested by using them along with modern samples from large lakes to reconstruct land cover using the REVEALS model (Sugita, 2007a) and comparing the obtained estimates with modern vegetation data. Second, for an evaluation of the soundness of the REVEALS reconstructions in terms of plant abundance, REVEALS-based quantitative reconstructions of past plant cover using fossil pollen records can be compared with other palaeoecological information, such as climate reconstructions, and archeological/historical data on human activity.

In order for future RPP study results to be more easily compared with this dataset, it is recommended that the methods used in the field, for preparing the vegetation input data, and running the ERV submodels are as standard as possible. The protocol for collection of pollen samples and vegetation surveys within 100 m proposed by Bunting et al. (2013) is a useful standardization of methods. It is also important to aim for a random distribution of the study sites in order to obtain as reliable RPP estimates as possible. Due to the practical limits of spatial precision in vegetation inventories and mapping, it is recommended to use vegetation data within rings of 5 to 10 meters to distance weight vegetation, rather than using a 1 m increment that requires a degree of precision in vegetation data that we cannot achieve. Finally, vegetation data should be extracted for a distance of 3 km around each sampling point given that RSAP estimates have been found to be in the range of 1–2 km in most studies performed so far in Europe and China. Smaller RSAP are found if the vegetation mosaic is particularly fine grained such as in cultural landscapes (Broström et al., 2005; Li et al., 2017).

## Author contributions

FL and M-JG designed the study and the paper, and interpreted the results in collaboration. FL wrote the first draft of the manuscript and made all Tables and Figures. M-JG corrected the manuscript, wrote additional text, and improved the language of the manuscript. QX provided much support to FL and M-JG for the Shandong study (Li et al., 2017), generously gave the permission to reanalyse the original data from two studies, and commented the manuscript. MB commented the manuscript in great detail and thoroughly improved and corrected the language. YL, JLi, and HM contributed data for the review. JLu, PZ, SZ, YZ, and WS were involved in two of the studies reviewed in the manuscript. QC commented the results and manuscript.

### Conflict of interest statement

The authors declare that the research was conducted in the absence of any commercial or financial relationships that could be construed as a potential conflict of interest.
