# Supplementary material for: A Review of Relative Pollen Productivity Estimates From Temperate China for Pollen-Based Quantitative Reconstruction of Past Plant Cover
Source: Front Plant Sci. 2018 Sep 5;9:1214. doi: 10.3389/fpls.2018.01214 (PMC6134201; doi:10.3389/fpls.2018.01214)
Supplement: Supplementary file 1 [file Table_1.docx]

Electronic Supplementary Material

**Title of the paper: A review of relative pollen productivity estimates from temperate China for pollen-based quantitative reconstruction of past plant-cover**

Furong Li^1^, Marie–José Gaillard^1^, Qinghai Xu^2^, M. Jane Bunting^3^, Yuecong Li^2^, Jie Li^2^, Huishuang Mu^2^, Jingyao Lu^2^, Panpan Zhang^2^, Shengrui Zhang^2^, Qiao–Yu Cui^4^, Yahong Zhang^5^ and Wei Shen^5^

^1^Department of Biology and Environmental Science, Linnaeus University, Kalmar SE–39182, Sweden,

^2^College of Resources and Environment Science, Hebei Normal University, Shijiazhuang 050024, China,

^3^School of Environmental Sciences, University of Hull, Cottingham Road, Hull, HU6 7RX, UK

^4^Institute of Geographic Sciences and Natural Resources Research, Chinese Academy of Sciences, Beijing, 100101, China

^5^ Institute of Nihewan Archaeology, Hebei Normal University, Shijiazhuang 050024, China

In this Electronic Supplementary Material we provide detailed descriptions of the study areas (chapter 1) and methods (chapter 2) used in the studies reviewed in the article.

**1. Description of the study areas**

Below we briefly describe the study areas in terms of vegetation types and flora. The location of the study areas (Figure 1) and related vegetation maps (Figures 2 and 3) are found in the published article. Other metadata for the study areas are found in Tables S1 and S2 in the Electronic Supplementary Material.

**1.1** **The Tibetan Plateau (Wang and Herzschuh, 2011. area a in Figures 1 and 2, Table S1)**

The Tibetan Plateau is characterized by elevations mostly exceeding 4000 m.a.s.l. The study area includes the alpine meadow zone and the alpine steppes in the eastern part of the Plateau. The major species in alpine meadows include *Kobresia pygmaea*, *K. humilis*, *K. capillifolia*, *Stipa purpurea* and *Polygonum sphaerastachyum*. These species are also common in the transition zone between meadows and steppes together with an abundance of *Artemisia gmelinii, A. argyi* and *Chenopodium hybridum*.

RPP was estimated for *Artemisia*, Poaceae, Chenopodiaceae and Cyperaceae, all dominant pollen taxa in fossil pollen assemblages from this region.

**1.2** **The Alashan Plateau, western Inner Mongolia (Li et al., 2011. area h in Figures 1 and 2, Table S1)**

The study area is located at elevations of 1000–1200 m a.s.l. west and east of Helan Mountains. Helan Moutains separate contrasting vegetation types and climate regimes, desert–steppe in the east with mean annual precipitations > 150 mm, and desert in the west with mean annual precipitations generally < 150 mm. The desert–steppe is dominated by *Stipa klemenzii*, *Cleistogenes keng*, *Artemisia frigida* and *Caragana tibetica*, while the desert is characterized by vegetation cover < 30% and the dominance of species of *Artemisia*, *Nitraria*, *Salsola*, *Suaeda*, *Reaumuria* and *Zygophyllum*. The vegetation near the Helan Mountains is semi–desert, with a 25–40% plant cover consisting of species of *Nitraria*, *Artemisia desterorum*, *Reaumuria soongorica* and *Stipa klemenzi*.

RPP was estimated for Poaceae, *Artemisia,* Chenopodiaceae, and *Nitraria*

- 1. **The Xinglong Mountains** (Wu et al., 2013. area i in Figure 1)

The study region is located at elevations of 1800–3600 m. a.s.l. The climate is temperate and semi–arid with mean annual precipitation of 450–700 mm and mean annual temperature of 3–7 ℃. The study region includes alpine shrub, alpine meadow, coniferous forest, mixed

Table S1. Metadata for the seven study regions (a-g, Figs. 1 and 2 in the published article) selected for the synthesized RPP datasets. See text for more explanations

| **see fig.1** | **Study area** | **Pollen sample type** | **References** | **Coordinates** | **Number of sites** | **Site distribution** | **Distance weighting method** | **ERV sub models** | **Major vegetation types sampled** | **Distance from sampling sites (m)** | | | **Vegetation survey method** | | |
| --- | --- | --- | --- | --- | --- | --- | --- | --- | --- | --- | --- | --- | --- | --- | --- |
|  |  |  |  |  |  |  |  |  |  | **Zone A** | **Zone B** | **Zone C** | **Zone A** | **Zone B** | **Zone C** |
| a | north eastern Tibetan Plateau | lake sediment | Wang and Herzschuh, 2011 | (33°–36°N, 96°30′–99°30′E) | 19 | selected | 1/d, 1/d2, Prentice, Sugita model | 1 and 2 | alpine meadow and steppe | None | None | 5000 | None | None | Vegetation map and literature |
| b | Shandong, eastern China | moss polsters | Li et al., 2017 | (35°–36°30′N, 117°–118°30′E) | 37 | random | 1/d, Prentice model | 1, 2 and 3 | semi-open agricultral landscape | 10 | 100 | 1500 | Crackles | Crackles | Land use map from satellite image |
| c | Taiyue Mountains | moss polsters | Zhang et al., 2017 | (36°33′N-36°54′N, 111°45′E-112°30′E) | 30 | random | Prentice model | 1, 2 and 3 | warm temperate deciduous broad-leaved forest | 10 | 100 | 5000 | Crackles | Crackles | Vegetation map |
| d | central Inner Mongolia | soil | Ge et al., 2015 | (41°–43°N, 114°–116°E) | 30 | random | 1/d, 1/d2, Prentice model | 1, 2 and 3 | steppe, meadow and cultivated | 10 | 100 | 1000 | Crackles | Crackles | Vegetation map |
| e | northeast Inner Mongolia | soil | Li et al., in prep. | (45°-47°N, 118°-119°30′E) | 30 | selected | Prentice model | 1 | steppe, meadow | 10 | 100 | 1000 | Crackles | Crackles | Vegetation map |
| f | Changbai Mountains, | moss polsters | Li et al., 2015 | (42°–42°30′N, 125°45′–126°45′E) | 20 | stratified random | Prentice model | 1, 2 and 3 | mixed broad leaved and coniferous forest | 10 | 100 | 5000 | Crackles | survey at 40m, 70m, and 100m N,S,E,W | Vegetation map |
| g | Changbai Mountains, | moss polsters | Zhang et al., 2017 | (42°15′-42°45′N, 127°45′-128°30′E) | 36 | random | Prentice model | 1, 2 and 3 | mixed broad leaved coniferous forest | 10 | 100 | 5000 | Crackles | Crackles | Vegetation map |

coniferous–deciduous forest, and deciduous forest vegetation. The major tree species are *Abies fargesii*, *Picea purpurea*, *P. crassifolia*, *P. wilsonii*, *Populus davidiana*, *Betula platyphylla*, *B. albo–sinensis*, *B. utilis* and *Quercus liaotungensis*. Shrubs belong mainly to *Corylus mandshurica*, *Ostryopsis davidiana*, *Rosa hugonis*, *Cotoneaster multiflorus*, *Berberis kansuensis*, *Syringa oblate*, *Hippophae rhamnoides*, *Salix oritreoha*, and *Rhododendron* species. Herbs consist primarily of *Deschampsia caespitosa*, *Carex atrofusca*, *Meconopsis* species, *Polygonum viviparum*, *P. macrophyllum*, *Saxifraga tangutica*, *Arenaria przewalskii* and *Androsace* species.

RPP was estimated for *Quercus, Picea, Betula, Hippophae, Artemisia,* Poaceae, and Cyperaceae.

- 1. **Central Inner Mongolia** (Xu et al., 2014. area m in Figure 1; Ge et al., 2015. area d in Figure 1; He et al., 2016. areas j, k and l in Figure 1; Han et al., 2017. area n in Figure 1)

The study region of Xu et al. (2014) is situated at elevations of 800–1400 m a.s.l. and the climate is arid and semi–arid temperate with mean annual temperature 0–1℃ and mean annual precipitation of 300–450 mm, respectively. Dominant vegetation communities are temperate grassland and shrub, some hygrophyte and halophyte meadows, and sparse semi- open *Ulmus pumila* woodland. The temperate grassland consists of *Stipa grandis, Leymus chinensis, Artemisia desertorum, Filifolium sibiricum, Stipa krylovii* and *Agropyron cristatum*. Meadows are dominated by Poaceae and Cyperaceae. The deciduous shrub communities include *Salix microstachya* and *Betula fruticosa* with an herb layer dominated by species of *Phragmites* and *Carex*.

RPP was estimated for Poaceae, Cyperaceae, *Artemisia*, Chenopodiaceae, *Potentilla* type, *Thalictrum*, Iridaceae, Brassicaceae, *Ulmus,* and *Ephedra*.

Table S2. Metadata for the eight study regions (h-o, Figs. 1 and 2 in the published article) not selected for the synthesized RPP datasets. See text for more explanations

| **see fig.2** | **Study area** | **Pollen sample type** | **References** | **Cordinates** | **Number of sites** | **Pollen sample distribution method** | **Distance weighting method** | **ERV sub models used** | **Major vegetation types sampled** | **Distance from sampling sites (m)** | | | | **Vegetation survey method** | | |
| --- | --- | --- | --- | --- | --- | --- | --- | --- | --- | --- | --- | --- | --- | --- | --- | --- |
|  |  |  |  |  |  |  |  |  |  | **Zone A** | | **Zone B** | **Zone C** | **Zone A** | **Zone B** | **Zone C** |
| h | Alashan Plateau, western Inner Mongolia | soil and trap | Li et al., 2011 | (38°–41°N, 104°45′–109°E) | 64 | selected | Prentice model | 1 and 2 | desert, semi-desert and desert steppe | 10 | 100 | | 1000 | estimates | vegetation map | vegetation map |
| i | Xinglong Mountains | soil | Wu et al., 2013 | (35°30′–36°N, 103°45′–104°15′E) | 30 | selected | Prentice model | 1 and 2 | forest, alpine meadow and alpine shrubs | 20 | 100 | | 2000 | vegetation survey | NA | NA |
| j | Sunitezuoqi, central Inner Mongolia | trap | He et al., 2016 | (43°30′–44°30′N, 113°–114°E) | 22 | selected | Prentice model | 1, 2 and 3 | desert | 20 | 100 | | 1000 | quadrats survey at distance of 0, 0.5, 1.5, 2.5, 4, 6, 8, 10, 15 and 20 m from two directions | survey at each 10 m follow the same direction as zone A | survey at each 100 m follow the same direction as zone A, from 1000, use the sattelite image |
| k | Xilinhaote, central Inner Mongolia |  |  | (43°–44°N, 116°–117°30′E) | 12 |  |  |  | steppe |  |  |  |  |  |  |  |
| l | Hulunbeier, east Inner Mongolia |  |  | (49°–50°N, 120°–121°E) | 23 |  |  |  | forest-steppe ecotone |  |  |  |  |  |  |  |
| m | central Inner Mongolia | soil | Xu et al., 2014 | (42°–44°N, 115°30′–117°30′E) | 30 | stratified random | 1/d, 1/d2, Prentice, Sugita model | 1 and 2 | temperate grassland, hygrophyte or halophyte meadow and desert | 10 | 100 | | 1000 | Crackles and vegetation map | random quadrats | vegetation map |
| n | Xilinguole, central Inner Mongolia | lake sediment | Han et al., 2017 | (42°–45°N, 112°45′–117°45′E) | 14 | selected | Sugita model | 1 and 2 | forest-steppe ecotone | None | within ca. 400 m from lake shore | | 5000 | None | 1m² quadrat surveys in each vegetation community identified along all directions N, S, E, etc…. | land use map from satellite image |
| o | Hulunbeier, north Inner Mongolia |  |  | (49°–49°30′N, 116°45′–120°45′E) | 10 |  |  |  |  |  |  |  |  |  |  |  |

The study area (area d in Figure 1) used in Ge et al. (2015) is located at elevations of 1300-1500 m a.s.l. in the Bashang grassland, which is characterized by steppe and meadow vegetation with patches of farmland. The climate is semi–arid temperate with mean annual temperature of 1–3 ℃ and mean annual precipitation of 300–400 mm. The dominant species are *Stipa capillata, Leymus chinensis, Achnatherum splendens, Cleistogenes squarrosa, Artemisia* spp., Chenopodiaceae, Compositae SF Asteroidae, *Potentilla*, Fabaceae, Cyperaceae and Convolvulaceae.

RPP was estimated for *Potentilla* type, Liliaceae, Lamiaceae, Fabaceae, Cyperaceae, Convolvulaceae, Chenopodiaceae, Compositae (named Asteraceae in the original paper), *Artemisia*, and Poaceae.

Two of the study areas reported in He et al. (2016) are located in central Inner Mongolia. Area j (Figure 1, main article) is located at elevations of 1000-1200 m a.s.l. in the desert-steppes ecotone of northwest Xilingguole characterized by mean annual precipitation of 150-200 mm and a mean annual temperature of 3 ℃, with vegetation dominated by *Stipa* spp. and *Caragana*. Area k (Figure 1) is located at elevations around 900–1300 m in the steppes of Xilinghaote where mean annual precipitation is <300 mm and mean temperature around 0-3 ℃. Here the vegetation is mainly composed of species of Poaceae, *Artemisia* and *Thymus.*

RPP was estimated for *Pinus, Quercus, Betula, Ulmus,* Poaceae, *Artemisia,* Chenopodiaceae, Compositae, and Cyperaceae

One of the study areas reported in Han et al. (2017) covers a broad region elevation changes from 1000–1700 m a.s.l. in the forest-steppe ecotone in Xilingguole, central Inner Mongolia. The climate in this area is semi-arid temperate with mean annual temperatures and precipitations of -3–5 ℃ and 200–400 mm, respectively. The vegetation is characterized by sparse patches of forest in the steppe region. Trees mainly include species of *Larix, Pinus, Quercus, Populus, Betula, and Ulmus*, while herbs are dominated by species of Poaceae, Chenopodiaceae, and Compositae.

RPP of *Artemisia*, Compositae, Poaceae, Chenopodiaceae and Cyperaceae were calculated in He et al. (2016).

- 1. **Northeastern Inner Mongolia (He et al., 2016. area l in Figure 1; Han et al., 2017. area o in Figure 1; Li et al., in prep. area e in Figure 1)**

One of the study areas reported in He et al. (2016. area l in Figure 1) is located at elevations around 600–900 m a.s.l. in northeastern Inner Mongolia. The mean annual temperature is around 0℃ and mean annual precipitation 250–350 mm. The vegetation in this area is characteristic of the forest-steppe ecotone. The main tree genera present are *Pinus* and *Betula*, and herbs are represented mainly by species of Poaceae and *Artemisia*.

RPP was calculated for *Artemisia*, Compositae, Poaceae, Chenopodiaceae and Cyperaceae.

A second study from northeastern Inner Mongolia is reported by Han et al. (2017. area o in Figure 1). Their study area is located at elevations around 500–700 m a.s.l. in the forest–steppe ecotone in Hulunbeier, northern Inner Mongolia. Mean annual precipitation is in the range 250-310 mm and mean annual temperatures between -1℃ and 1℃. The main tree genera are *Ulmus* and *Pinus*, with temperate deciduous *Salix* species dominating the shrub layer*.* The main herbaceous taxa are species of Poaceae, Chenopodiceae, Compositae and Cyperaceae.

RPP was estimated for *Pinus, Quercus, Betula, Ulmus, Poaceae, Artemisia,* Chenopodiaceae, Compositae, and Cyperaceae using the pollen and vegetation data from Hulunbeier and Xilingguole (central Inner Mongolia, see above) together.

The study area of Li et al. (in prep. area e in Figure 1) is characterized by steppe and meadow vegetation at altitudes of 830–1450 m a.s.l. The climate is arid and semi-arid temperate. Mean annual precipitation is in the range 250–310 mm and mean annual temperatures between -1℃ and 1℃. The main plant species recorded include *Stipa baicalensis*, *S. grandis*, *S. krylovii*, *Leymus chinensis*, *Cleistogenes squarrosa*, and *Galium verum*. Other common taxa are *Hemerocallis dumortierii, Potentilla flagellaris, P. bifurca, Sanguisorba officinalis, Polygonum tortuosum, Artemisia frigida, A. canacetifolia,* and *Chenopodium glaucum*.

RPP was estimated for Poaceae, *Sanguisorba*, *Artemisia*, Chenopodiaceae, *Mentha* type (named *Thymus* in the original paper), Caryophyllaceae, and *Stellera*.

**1.6. Taiyue Mountains (Zhang et al., 2017. area c in Figure 1)**

The Taiyue Mountains are located in the central and southern parts of Shanxi province. The climate is warm temperate with mean annual temperature c. 8.5 ℃ and mean annual precipitation ca. 660 mm. The characteristic vegetation in the study zone between elevations of 1100–1800m a.s.l. is broadleaf-conifer forest with *Pinus tabulaeformis*, *Larix principisrupprechtii*, *Quercus liaotungensis*, *Populus davidiana*, *Betula platiphylla*, and *Juglans mandshurica*. *Larix principisrupprechtii* forest is dominant at elevations of 1800–2200m. Above 2200m, trees are replaced by species-rich shrub meadows, especially the shrub species *Hippophae rhamnoides*, *Lespedeza bicolor*, *Rosa xanthina*, *Lonicera japonica*, *Rhododendron micranthum*, and *Syringa reticulate*, and herb species belonging to *Carex*, *Artemisia*, *Thalictrum*, *Agrimonia*, and Compositae SF. Asteroideae*.*

RPP was estimated for *Pinus, Quercus, Juglans, Betula, Larix*, Ranunculaceae, *Artemisia*, Poaceae, Elaeagnaceae and Cyperaceae.

**1.7. Changbai Mountains (Li et al., 2015. area f in Figure 1; Zhang et al., 2017. area g in Figure 1)**

The Changbai Mountains are characterized by a mosaic of various types of broadleaved, coniferous, and mixed broadleaved-coniferous forests. The two studies so far reported focus on the elevation range 500-1700m, where mean annual temperature is between 2–6 ℃ and mean annual precipitation 600–800 mm. The major tree species include *Quercus mongolica*, *Quercus liaotungens*, *Juglans mandshurica*, *Tilia mandshurica*, *Pinus koraiensis*, *Acer mono*, *Larix olgensis*, *L. gmelinii*, *Betula platyphylla*, *B. ovalifolia*, and *Ulmus laciniata*. The aim of the study of Li et al. (2015) was to obtain RPP estimates for the dominant tree taxa in the three major woodland types of the Changbai Mountains, i.e. broadleaved, coniferous, and mixed broadleaved-coniferous woodlands.

RPP was estimated for *Pinus*, *Quercus, Juglans, Betula, Fraxinus, Ulmus, Tilia,* and *Larix.*

A second study in the area focused on the northern part of the mountains (Zhang et al., 2017). There mixed broadleaved-conifer forest is the major woodland type at elevations below 1100m a.s.l.. It is dominated by *Pinus koraiensis* and includes *Larix olgensis*, *Abies holophylla*, *Taxus chinensis*, and *Picea jezoensis.* Coniferous forest grows at elevations of 1100–1700m a.s.l., and is characterized by *Picea*, *Abies*, and *Pinus koraiensis,* with shrubs and herbs such as *Corylus mandshurica*, *Philadelphus incanus*, *Lonicera ruprechtiana*, *Anisodus acutangulus*, *Filipendula palmate*, *Phryma leptostachya*, and species of *Carex, Artemisia,* and Compositae SF Asteroidae.

RPP was estimated for *Pinus*, *Quercus*, *Juglans, Betula, Fraxinus, Ulmus*, *Tilia*, *Larix*, *Artemisia*, Poaceae and Cyperaceae.

**1.8. The cultural landscapes of Shandong province (Li et al., 2017. area b in Figure 1)**

The study was conducted in the low mountain regions of central and southern Shandong at elevations of 100-500m a.s.l.. Vegetation is strongly impacted by agriculture both on the mountain slopes (terraces) and in the plains (mostly large fields under modern agricultural practices). Ancient terrace systems are still well preserved in most of the province and partly cultivated with traditional practices. In this environment, plant species of fallow and ruderal land and wood patches are still common. The cultivated terraces are generally replaced by grazing land and woods in the upper part of the mountains. The terraces are used for crops such as *Arachis hypogaea*, *Ipomoea batatas*, and cereals, and for cultivation of *Prunus cerasus*, *Juglans regia, Castanea mollissima,* *Crataegus pinnatifida*, and *Lonicera japonica*. Ruderals include mainly *Artemisia annua*, *A. sacrorum*, Caryophyllaceae, Brassicaceae, and numerous species of Compositae. The terrace walls are characterized by a species-rich flora where *Lespedeza bicolor*, *Humulus scandens*, and species of *Artemisia* and other Compositae are common. Ruderal herb species and shrubs such as *Vitex negundo* are common in fallow land. *Pinus tabulaeformis, P. thunbergii, Platycladus orientalis, Quercus variabilis*, *Q. acutissima,* and *Robinia pseudoacacia* occur as isolated trees or in woodland patches of various sizes. *Platycladus orientalis* is often planted. Grazing land is dominated by species of Poaceae often accompanied by a species-rich forb flora.

RPP was estimated for *Castanea, Pinus, Quercus, Ulmus, Juglans*, Cupressaceae, *Robinia*/*Sophora*, *Vitex negundo*, *Aster/Anthemis* type, *Cannabis/Humulus*, Caryophyllaceae, Cyperaceae, Amaranthaceae/Chenopodiaceae, *Artemisia*, Brassicaceae, *Galium* type, Poaceae, and Compositae SF. Cichorioideae.

**2. Methods**

**2.1 Site selection**

Among the twelve RPP studies reviewed in this paper, only Ge et al. (2015), Li et al. (2017) and Zhang et al. (2017. Taiyue Mountains study area) used true random sample points in their studies (Fig. 2 in the article).

In the other studies, sites were selected in order to achieve a more or less even number of sites per vegetation type represented in the study area, which is a type of stratified random selection (Fig. 2 in the article), or site selection was governed by the location of lakes in the case of pollen data collected from lake surface sediments (Tibetan Plateau, Wang and Herzschuh, 2011; central and northeast Inner Mongolia, Han et al., 2017; Figure 3 in the main text). In some cases the distribution of sites followed a series of transects through the study area and samples were collected every 30–40 km (e.g. on the Alashan Plateau, western Inner Mongolia, Li et al., 2011; Fig. 3 in the article). He et al. (2016) used pollen traps but do not specify their distribution within the study area (Fig. 3 in the article). In central Inner Mongolia, sites were selected in the largest patches of major vegetation types and avoided areas with human impact (Xu et al., 2014; Figure 2 in the article). In the Changbai Mountains (Li et al. 2015), sites were selected in large openings within the forest (Fig. 2 in the article), analogous to large mires/bogs in terms of pollen deposition basin, which implies a larger source area of pollen than moss polsters within the forest.

**2.2 Pollen and vegetation data**

Pollen was extracted from moss polsters (Wu et al., 2013; Li et al., 2015; Li et al., 2017; Zhang et al., 2017), lake surface sediments (Wang and Herzschuh, 2011; Han et al., 2017), surface soils (Li et al, 2011; Xu et al., 2014; Ge et al., 2015; Li et al., in prep.) and pollen traps (part of Li et al., 2011; He et al., 2016). All moss polsters were collected according to the protocol of Bunting et al. (2013) except for the study of Wu et al. (2013) where pollen samples consisted of a mixture of several moss polsters collected within a 10m x 10m area. Lake surface sediments from the Tibetan Plateau (Wang and Herzschuh, 2011) were collected in 19 lakes with a radius of ca. 100m, and from Inner Mongolia in lakes with various radius (Han et al., 2017). In Inner Mongolia (He et al., 2016), pollen traps were placed at fixed locations in the field and collected after 3 and 6 years.

In all study areas, vegetation surveys in the field were combined with extraction of vegetation data from vegetation maps or satellite pictures to obtain the necessary information to calculate distance weighted plant abundance. However, the methods used differ between studies in terms of field survey methods and distances (generally up to 100 m around the pollen sample), the source of the vegetation data beyond 100m, and the spatial resolution of both field surveys and data extraction beyond 100m (Table S1 and S2).

Bunting et al. (2013) proposed a standard protocol for pollen and vegetation data collection with the aim of obtaining RPP estimates that can be compared between studies. This protocol states that vegetation data should be collected in the field following specified methods in two spatially well-defined zones, i) 0–10 m from the pollen sample (survey of a defined network of 21 1m^2^ quadrats) and ii) 10–100m from the pollen sample (vegetation mapping and surveys of random quadrats and along random lines). This protocol was used in most of the studies reviewed here except those by Wang and Herzschuh (2011), Li et al. (2011), Wu et al. (2013), Han et al. (2017) and He et al. (2016). The methods of vegetation survey in the 10–100m zone also differed from the protocol in the studies of Xu et al. (2014) and Li et al (2015). Xu et al. (2014) carried out the vegetation surveys by using 1m^2^ quadrats randomly distributed along four lines following the four directions NE, SE, NW, NE. Li et al. (2015) performed vegetation surveys by positioning 12 quadrats at 40m, 70m and 100m from the center of the sampling sites along the four cardinal directions, N, E, S, and W. The size of the quadrats was 1m^2^ in open vegetation, 16m^2^ in semi–open areas, and 100m^2^ (20mx5m) in forest vegetation.

For the area beyond 100m, existing maps or remote sensing methods were used. Wang and Herzschuh (2011) used the vegetation atlas of China (1:1,000,000) (Hou, 2001) for the spatial distribution of communities, and their taxa composition was assessed using both field surveys (Kürschner et al., 2005; the number and breast–height diameter of all tree species were recorded in 40 m^2^ quadrats, and percentage cover of all shrubs and herbs were recorded in 4m^2^ and 1m^2^ quadrats, respectively) and published vegetation surveys (Zhou et al., 1986; Wu, 1995; Wang et al., 2006; and Miehe et al., 2008).

On the eastern Alashan Plateau (Li et al., 2011), the percentage cover of target taxa was estimated for a radius of 10m around each site, and the vegetation Atlas of China (Hou, 2011) was used to extract vegetation data between 10m and 1000m.

Beyond 100m, vegetation data was extracted from existing vegetation maps in all studies, e.g. the 1:1,000,000 vegetation Atlas of China (Hou, 2011) and a 1:25000 scale forest map (Zhang, 2007) in Li et al. (2015), with the exception of Li et al. (2017) who created vegetation maps for an area of 1500m radius around the pollen sites from satellite images (Google Earth Professional) using maximum likelihood classification in ArcView. The plant composition in each mapped vegetation unit was estimated from the vegetation surveys within 100m (see Li et al., 2017 for details).

**2.3 Fall speed of pollen** (Table S3)

A second important taxon–specific parameter is the fall speed of each pollen type (FSP). This was calculated in all studies using Stoke’s law for the terminal velocity of small particles and measurements of the size of pollen grains (Gregory, 1973). Values for the density of the pollen grains and atmospheric conditions (density and viscosity of the air in an ordinary surface temperature and pressure) are also needed for this calculation. The density used for the pollen grains is generally taken from the literature (e.g. reviews of Gregory, 1973; Jackson and Lyford, 1999). For taxa without available direct measurements, the density is usually set to 1 for non–saccate grains and 0.5 for saccate grains (e.g. Li et al., 2017). Stoke’s law is adequate for circular particles, and Falck’s correction can be applied for ellipsoid particles such as Apiaceae, Cyperaceae, *Pinus*, and *Picea*. Information on density values used and calculation methods is only specified by Li et al. (2017), Zhang et al. (2017), and Li et al. (in prep.).

**2.4 ERV model runs, reference taxon, and RSAP estimate**

The distance weighting methods and ERV sub–models used in ERV modelling vary between studies, and where several combinations were tested, the vegetation distance weighting method and ERV submodel considered to provide the best results by the authors also varies. For example, Xu et al., (2014) used all four common vegetation distance weighting methods (1/d, 1/d^2^ and the Prentice’s taxon-specific and –Sugita’s ring source method) with ERV submodels 1 and 2, and identified the results from submodel 2 as the most reliable based on the values of the likelihood function scores (these scores were lowest when submodel 2 was used, regardless of taxon-specific or ring source distance weight method were used,therefore the fit between raw and corrected data were best). Li et al. (2015) and Ge et al. (2015) compared all three ERV submodels but only used the Prentice–Sugita distance weighting method, and identified the results from submodel 3 as producing the best fit scores. Ge et al. (2015), Li et al. (2017) and Zhang et al. (2017) compared all three ERV submodels for both 1/d and Prentice distance-weighting methods and all found that the combination of Prentice’s taxon-specific distance weighting and submodel 3 yielded the best fit. Li et al. (2017) used absolute plant cover as their measure of vegetation due to the large amounts of bare ground (in m^2^/m^2^) and, therefore, the outputs of submodel 3 are expected theoretically to be the most reliable.

Estimates of pollen productivity calculated using the ERV approach are expressed relative to a reference taxon (which has its pollen productivity set to 1). In theory any taxon can serve as the reference taxon, but for the best analysis results the reference taxon needs to be present in both pollen and vegetation data at many of the sites used in the analysis, have a wide range of values of both pollen and vegetation, and have intermediate pollen productivity. In most RPP studies to date Poaceae has been used as the reference taxon in semi open and open landscapes, and *Quercus* in wooded landscapes. Poaceae was selected as the reference taxon in six of the Chinese studies (Wang and Herzschuh, 2011; Xu et al., 2014; Ge et al., 2015; Li et al., 2017; Han et al., 2017; Li et al. in prep.), *Quercus* in three studies (Wu et al., 2013; Li et al., 2015; Zhang et al., 2017), Chenopodiaceae in one study (Li et al., 2011), and *Artemisia* in one study (He et al., 2016).

We chose to use Poaceae as the reference taxon for all studies in order to compare the RPP values between studies. Published values of RPP*_Quercus_*, RPP_Chenopodiaceae_, and RPP*_Artemisia_* were converted to RPP_Poaceae_ for comparison. Poaceae is most commonly chosen as reference taxon because it is very common in the pollen assemblages and generally exhibits good gradients of pollen and vegetation data and a close to linear pollen-vegetation relationship before correction with the ERV model. However, Poaceae is a pollen taxon which includes a large number of species, and the species often differ between study areas, which may result in

Table S3. Values of fall speed of pollen (FSP) used in the eleven studies reviewed in this paper and mean FSP based on all values.

| **TAXON** | **Wang and Herzschuh, 2011** | **Li et al., 2011** | **Wu et al., 2013** | **Xu et al., 2014** | **Li et al., 2015** | **Ge et al., 2015** | **Li et al., 2017** | **Zhang et al., 2017** | **Zhang et al., 2017** | **Li et al., in prep.** | **Han et al., 2017** | **He et al., 2016** | **mean FSP for REVEALS** | **European fall speeds** |
| --- | --- | --- | --- | --- | --- | --- | --- | --- | --- | --- | --- | --- | --- | --- |
| *Pinus* |  |  |  |  | 0.039 |  | 0.028 | 0.03 | 0.041 |  | 0.039 |  | 0.035 | 0.031 |
| *Cupressaceae* |  |  |  |  |  |  | 0.01 |  |  |  |  |  | 0.01 | 0.016 *Juniperus* |
| *Robinia/Sophora* |  |  |  |  |  |  | 0.022 |  |  |  |  |  | 0.022 |  |
| *Quercus* |  |  | 0.018 |  | 0.018 |  | 0.025 | 0.016 | 0.019 |  | 0.018 |  | 0.019 | 0.035 |
| *Juglans* |  |  |  |  | 0.03 |  | 0.037 | 0.028 | 0.031 |  |  |  | 0.032 | 0.057 *Fagus* |
| *Betula* |  |  | 0.011 |  | 0.019 |  |  | 0.011 | 0.017 |  | 0.019 |  | 0.015 | 0.024 |
| *Castanea* |  |  |  |  |  |  | 0.004 |  |  |  |  |  | 0.004 |  |
| *Tilia* |  |  |  |  | 0.03 |  |  |  | 0.027 |  |  |  | 0.029 | 0.032 |
| *Ulmus* |  |  |  | 0.01 | 0.022 |  | 0.032 |  | 0.019 |  | 0.022 |  | 0.021 | 0.032 |
| *Larix* |  |  |  |  | 0.135 |  |  | 0.117 |  |  |  |  | 0.126 |  |
| *Fraxinus* |  |  |  |  | 0.017 |  |  |  |  |  |  |  | 0.017 | 0.022 |
| *Vitex negundo* |  |  |  |  |  |  | 0.016 |  |  |  |  |  | 0.016 |  |
| *Nitraria* |  | *0.016* |  |  |  |  |  |  |  |  |  |  | 0.016 |  |
| *Elaeagnaceae* |  |  |  |  |  |  |  | 0.012 |  |  |  |  | 0.012 |  |
| *Poaceae* | *0.019* | *0.023* | 0.034 | 0.016 |  |  | 0.022 | 0.02 | 0.017 | 0.017 | 0.022 | 0.032 | 0.022 | 0.035 |
| *Artemisia* | *0.01* | *0.01* | 0.01 | 0.009 |  | 0.009 | 0.015 | 0.007 | 0.009 | 0.009 | 0.007 | 0.021 | 0.011 | 0.025 |
| *Cyperaceae* | *0.029* |  | 0.023 | 0.015 |  |  | 0.037 | 0.019 | 0.019 | 0.014 | 0.017 | 0.028 | 0.022 | 0.035 |
| *Amaranth./Chenop.* | *0.012* | *0.009* |  | 0.011 |  | 0.01 | 0.027 | 0.012 | 0.011 | 0.011 | 0.009 | 0.027 | 0.014 | 0.019 |
| *Ranunculaceae* |  |  |  |  |  |  |  | 0.007 |  |  |  |  | 0.007 | 0.019 |
| *Brassicaceae* |  |  |  | 0.003 |  |  | 0.02 |  |  |  |  |  | 0.012 |  |
| *Thalictrum* |  |  |  | 0.007 |  |  |  |  |  | 0.013 |  |  | 0.01 |  |
| *Potentilla type* |  |  |  | 0.007 |  | 0.012 |  |  |  |  |  |  | 0.01 | 0.018 |
| *Iridaceae* |  |  |  | 0.012 |  |  |  |  |  |  |  |  | 0.012 |  |
| *Compositae* |  |  |  | 0.012 |  | 0.019 | 0.026 |  |  |  | 0.017 | 0.019 | 0.019 |  |
| *Ephedra* |  |  |  | 0.015 |  |  |  |  |  | 0.013 |  |  | 0.014 |  |
| *Convolvulaceae* |  |  |  |  |  | 0.043 |  |  |  |  |  |  | 0.043 |  |
| *Fabaceae* |  |  |  |  |  | 0.012 | 0.022 |  |  |  |  |  | 0.017 |  |
| *Lamiaceae* |  |  |  |  |  | 0.012 |  |  | 0.019 | 0.014 |  |  | 0.015 |  |
| *Liliaceae* |  |  |  |  |  | 0.013 |  |  | 0.014 |  |  |  | 0.014 |  |
| *Aster/Anthemis*type |  |  |  |  |  |  | 0.025 |  |  |  |  |  | 0.025 | 0.029 *Anthemis* |
| *Caryophyllaceae* |  |  |  |  |  |  | 0.039 |  |  | 0.022 |  |  | 0.031 |  |
| *Comp. SF. Cich.* |  |  |  |  |  |  | 0.028 |  |  |  |  |  | 0.028 | 0.051 |
| *Galium* type |  |  |  |  |  |  | 0.01 |  |  |  |  |  | 0.01 | 0.019 Rubiaceae |
| *Cannabis/Humulus* |  |  |  |  |  |  | 0.01 |  |  |  |  |  | 0.01 |  |
| *Cirsium* type |  |  |  |  |  |  | 0.075 |  |  |  |  |  | 0.075 |  |
| *Polygonaceae* |  |  |  |  |  |  |  |  | 0.019 | 0.019 |  |  | 0.019 |  |
| *Sanguisorba* type |  |  |  |  |  |  |  |  |  | 0.012 |  |  | 0.012 |  |
| *Stellera* |  |  |  |  |  |  |  |  |  | 0.009 |  |  | 0.009 |  |
| *Mentha* type (*Thymus*) |  |  |  |  |  |  |  |  |  | 0.014 |  |  | 0.014 |  |
| *Picea* |  |  | 0.132 |  |  |  |  |  |  |  |  |  | 0.132 |  |
| *Hippophae* |  |  | 0.015 |  |  |  |  |  |  |  |  |  | 0.015 |  |

different RPPs between sites even if the actual pollen production of the other taxa remain the same. *Quercus* as a reference taxon might be less problematic than Poaceae since the number of species involved is lower, although the species present also differ between regions of the world. This has not been formally tested to date, but it has to be kept in mind as one of the possible factor that might explain discrepancies in RPP between sites for a given taxon (e.g. Broström et al., 2008; Bunting et al, 2013).

Relevant Source Area of Pollen (RSAP) can be estimated on the basis of the curve of likelihood function scores or log likelihood, either by visual inspection or, for a more objective estimate, using a moving–window linear regression analysis (Sugita in Gaillard et al., 2008). The latter method tests whether the slope of the distance window is significantly different from zero. The middle of the first window in which the slope is not different from zero for a chosen p value is taken as the estimate of the RSAP. Li et al. (2017) uses this method, and all other studies reviewed use visual inspection of the curves of likelihood function scores or log likelihood.

**References**

Broström, A., Nielsen, A. B., Gaillard, M.-J., Hjelle, K., Mazier, F., Binney, H., Bunting, J., Fyfe, R., Meltsov, V., Poska, A., Räsänen, S., Soepboer, W., von Stedingk, H., Suutari, H., & Sugita, S. 2008. Pollen productivity estimates of key European plant taxa for quantitative reconstruction of past vegetation: a review. ***Vegetation History and Archaeobotany***, 17(5): 461–478.

Bunting, M. J., Schofield, J. E., & Edwards, K. J. 2013. Estimates of relative pollen productivity (RPP) for selected taxa from southern Greenland: A pragmatic solution. ***Review of Palaeobotany and Palynology***, 190: 66–74.

Gaillard, M.–J., Sugita, S., Bunting, M. J., Middleton, R., Broström, A., Caseldine, C., Giesecke, T., Hellman, S. E. V., Hicks, S., Hjelle, K., Langdon, C., Nielsen, A. B., Poska, A.,von Stedingk, H. & Veski, S. 2008. The use of modelling and simulation approach in reconstructing past landscapes from fossil pollen data: a review and results from the POLLANDCAL network. ***Vegetation History and Archaeobotany***, 17(5): 419–443.

Gregory, P. H. 1973. Spores: Their Properties and Sedimentation in Stiil Air. ***Microbiology of the atmosphere***.2: 15–29.

Ge, Y.-W., Li, Y.-C., Li, Y., Yang, X.-L., Zhang, R.-C., Xu,Q.-H. 2015. Relevant source area of pollen and relative pollen productivity estimates in Bashang steppe. ***Quaternary Sciences*** 2015: 934-945 (In Chinese with English abstract).

Han, Y., Liu, H.-Y., Hao, Q., Liu, X., Guo, W.-C. & Shangguan, H.-L. 2017. More reliable pollen productivityestimates and relative source area of pollen in a forest-steppe ecotone with improved vegetation survey. ***The Holocene***, 27(10): 1567-1577.

He, F., Li, Y.-Y., Wu, J., Xu., Y.-Z. 2016. A comparison of relative pollen productivity from forest steppe, typical steppe and desert steppe in Inner Mongolia. ***Chin Sci Bull***, 61: 3388–3400 (In Chinese with English abstract).

Hou, X.-Y. (2001). Vegetation Altas of China. Science Press, Beijing.

Jackson, S. T., & Lyford, M. E. 1999. Pollen dispersal models in Quaternary plant ecology: Assumptions, parameters, and prescriptions. The Botanical Review, 65(1): 39–75.

Kürschner, H., Herzschuh, U., Wagner, D., 2005. Phytosociological studies in the northeastern TP (NW China) — a first contribution to the subalpine scrub and alpine meadow vegetation. ***Botanische Jahrbücher der Systematik*** 126, 273–315.

Li, F.-R., Gaillard, M.–J., Sugita, S., Mazier, F., Xu, Q.-H., Zhou, Z.-Z., Zhang,Y.-Y., Li, Y.-C., & Laffly, D. 2017. Relative pollen productivity estimates for major plant taxa of cultural landscapes in central eastern China. Vegetation History and Archaeobotany, 26(6): 587–605.

Li, Y.-C., Bunting, M. J., Xu, Q.-H., Jiang, S.-X., Ding, W., & Hun, L.-Y. 2011. Pollen–vegetation–climate relationships in some desert and desert–steppe communities in northern China. ***The*** ***Holocene***, 21(6): 997–1010.

Li, Y., Nielsen, A. B., Zhao, X.-Q., Shan, L.-J., Wang, S.-Z., Wu, J., & Zhou, L.-P. 2015. Pollen production estimates (PPEs) and fall speeds for major tree taxa and relevant source areas of pollen (RSAP) in Changbai Mountain, northeastern China. ***Review of Palaeobotany and Palynology***, 216: 92–100.

Miehe, G., Miehe, S., Kaiser, K., , Liu, J.-Q. & Zhao, X.-Q. 2008. Status and Dynamics of the Kobresia pygmaea Ecosystem on the Tibetan Plateau. ***Ambio***, 37(4): 272–279.

Wang, W.-Y., Wang, Q.-J., Li, S.-X., Wang, G., 2006. Distribution and species diversity of plant communities along transect on the northeastern Tibetan Plateau. ***Biodiversity and Conservation*** 15, 1811–1828.

Wang, Y.-B., & Herzschuh, U. 2011. Reassessment of Holocene vegetation change on the upper Tibetan Plateau using the pollen–based REVEALS model. ***Review of Palaeobotany and Palynology***, 168(1): 31–40.

Wu, J., Ma, Y.-Z., Sang, Y.-L., Meng, H.-W., Hu, C.-L. 2013. Quantitative reconstruction of palaeovegetation and development R-values model: An application of R-value and ERV model in Xinglong Mountain natural protection region. ***Quanternary Research*** 33: 3, 554-564.(In Chinese with English abstract).

Wu, Z.-Y., chief editor, 1995. The vegetation of China. Science Press, Beijing. (in Chinese).

Xu, Q.-H., Cao, X.-Y., Tian, F., Zhang, S.-R., Li, Y. , Li, M.-Y., Li, J., Liu, Y. -L., & Liang, J. 2014. Relative pollen productivities of typical steppe species in northern China and their potential in past vegetation reconstruction. ***Science China Earth Sciences***, 57(6): 1254–1266.

Zhang, X.-S. Vegetation Map of the Republishing of China (1:1000000), 2007. Beijing: Geological Publishing House. (in Chinese).

Zhang, P.-P., Xu, Q.-H., Gaillard, M.-J., Mu, H.-S., Zhang, Y.-H., Lu, J.-Y. 2017. Research of main plant species relative pollen productivities and relevant source area of temperate coniferous and broad–leaved mixed forest in northern China. Quaternary sciences, 37(6): 1429–1443. (in Chinese with English abstract)

Zhou, X.-M., Wang, Z.-B., Du, Q., editor, 1986. Vegetation of Qinghai. People's Press of Qinghai, Xi'ning. (in Chinese).
